# Post-weaning Social Isolated Flinders Sensitive Line Rats Display Bio-Behavioural Manifestations Resistant to Fluoxetine: A Model of Treatment-Resistant Depression

**DOI:** 10.3389/fpsyt.2021.688150

**Published:** 2021-11-10

**Authors:** Khulekani Mncube, Marisa Möller, Brian H. Harvey

**Affiliations:** ^1^Centre of Excellence for Pharmaceutical Sciences (PharmaCen), Division of Pharmacology, School of Pharmacy, North-West University, Potchefstroom, South Africa; ^2^South African Medical Research Council Unit on Risk and Resilience in Mental Disorders, Department of Psychiatry and Mental Health and Neuroscience Institute, University of Cape Town, Cape Town, South Africa

**Keywords:** social anxiety, gene-environment model, risk and resilience, monoamines, inflammation, bipolar disorder

## Abstract

Treatment-resistant depression (TRD) complicates the management of major depression (MD). The underlying biology of TRD involves interplay between genetic propensity and chronic and/or early life adversity. By combining a genetic animal model of MD and post-weaning social isolation rearing (SIR), we sought to produce an animal that displays more severe depressive- and social anxiety-like manifestations resistant to standard antidepressant treatment. Flinders Sensitive Line (FSL) pups were social or isolation reared from weaning [postnatal day (PND) 21], receiving fluoxetine (FLX) from PND 63 (10 mg/kg × 14 days), and compared to Sprague Dawley (SD) controls. Depressive-, anxiety-like, and social behaviour were assessed from PND 72 in the forced swim test (FST) and social interaction test (SIT). Post-mortem cortico-hippocampal norepinephrine (NE), serotonin (5-HT), and dopamine (DA), as well as plasma interleukin 6 (IL-6), tumour necrosis factor alpha (TNF-α), corticosterone (CORT), and dopamine-beta-hydroxylase (DBH) levels were assayed. FSL rats displayed significant cortico-hippocampal monoamine disturbances, and depressive- and social anxiety-like behaviour, the latter two reversed by FLX. SIR-exposed FSL rats exhibited significant immobility in the FST and social impairment which were, respectively, worsened by or resistant to FLX. In SIR-exposed FSL rats, FLX significantly raised depleted NE and 5-HT, significantly decreased DBH and caused a large effect size increase in DA and decrease in CORT and TNF-α. Concluding, SIR-exposed FSL rats display depressive- and social anxiety-like symptoms that are resistant to, or worsened by, FLX, with reduced plasma DBH and suppressed cortico-hippocampal 5-HT, NE and DA, all variably altered by FLX. Exposure of a genetic animal model of MD to post-weaning SIR results in a more intractable depressive-like phenotype as well as changes in TRD-related biomarkers, that are resistant to traditional antidepressant treatment. Given the relative absence of validated animal models of TRD, these findings are especially promising and warrant study, especially further predictive validation.

## Introduction

While major depression (MD) affects approximately 216–322 million people worldwide (1), up to a third are non-responsive to an antidepressant (2) with up to a half failing to reach remission (3). In such cases of treatment-resistant depression (TRD), approximately 30% remain non-responsive to treatment after several treatment interventions (4) with that number decreasing with subsequent trials (5).

The factors contributing toward the development of TRD include (1) late age of onset of MD and family history (6); (2) non-response to first antidepressant (7); (3) history of abuse (sexual, physical, neglect) (8); (4) personality traits (9) and personality disorder (10); (5) comorbid psychiatric disorders (especially anxiety), insomnia, pain sensitivity, and gender (11); (6) current risk of suicide (7); (7) high recurrence rates (12); (8) and undetected psychotic symptoms (13). The treatment of MD has traditionally targeted monoaminergic systems by either blocking monoamine degradation or monoamine reuptake sites (14). Fluoxetine (FLX) is a selective serotonin reuptake inhibitor (SSRI) widely regarded as a standard-of-care treatment for MD and various anxiety disorders (15). Mechanistically, FLX increases serotonin (5-HT) while moderately increasing frontocortical and hypothalamic norepinephrine (NE) and dopamine (DA) (16).

Compared to MD patients, TRD has been associated with decreased dopamine-β-hydroxylase (DBH) (17), as well as hypothalamic-pituitary-adrenal (HPA)-axis hyperactivity, abrogated negative feedback and hypercortisolemia (18). Pro-inflammatory cytokines, interleukin (IL)-6 and tumour necrosis factor (TNF)-α, are also strongly implicated in the pathogenesis of MD, as well as being indicators and predictors of TRD (19, 20). Concerning the monoamines, reduced dopamine (DA) neurotransmission is especially implicated in TRD (21, 22), although elevated DA in cerebrospinal fluid and plasma is typically observed in TRD with psychotic features (23). Animal models have similarly shown depleted prefrontocortical DA (17), as well as increased mesolimbic ventral tegmental dopaminergic activity (24). Serotonin deficits in fronto-limbic sites (25) and inefficient NE neurotransmission is also associated with TRD (26). Neuro-anatomically, frontocortical dysfunction is postulated to underlie much of the cognitive and negative affect evident in MD, including anxiety and social deficits. Deficits in cognition, affect and volition are typically of hippocampal origin (27).

The development of MD is influenced by genetic susceptibility as well as adverse environmental factors (28). The Flinders Sensitive Line (FSL) rat displays behaviour akin to depressed humans, and is a useful genetic animal model with broad face, construct, and predictive validity for MD (29, 30). It shows broad response to various antidepressants following chronic dosing (29). Childhood adversity is known to be prodromal in the development of MD and TRD (8, 31). In animal studies, removing young rodents from their colony at weaning induces long-lasting behavioural changes that include neophobia, social withdrawal, disordered social interaction, and aggression (32). Early-life neurodevelopmental changes that parallel the development of MD can be modelled in rodents by employing post-weaning social isolation rearing (SIR). This preparation models early-life neurodevelopmental changes that parallel the development of MD (32), anxiety (33) and schizophrenia (psychosis) (34).

TRD models should include a phenotypic risk for MD, stress sensitivity, non-response to antidepressants, and response to TRD treatments (35). Current TRD models explore HPA-axis hyper-responsivity (35), gene-x-stress/environment models (36), the Wistar Kyoto rat model (37), and maternal separation (38). Importantly, multiple pre- and post-natal adverse events predict anxiety and psychiatric disorders (34, 39). Regarding TRD, clinical studies associate treatment resistance with prior adverse events (8). Indeed, exposing FSL rats to severe stress with reminders not only amplifies depressive-like behaviour, but shows resistance to imipramine (36). Thus, current thinking would suggest that a second post-natal hit interacts with pre-existing genetic factors to trigger or aggravate behavioural symptoms that eventually drive the development of treatment-resistance (36, 40).

Based on the above premise, this study aimed to develop a gene-x-environmental model of TRD by exposing FSL rats to post-weaning SIR and to evaluate antidepressant response in the resulting model. We hypothesised that a more severe depressive-like profile would ensue with more pronounced social impairments, both showing resistance to FLX treatment. Further, we hypothesised that biochemical changes commensurate with TRD would co-present with treatment resistance, including reduced cortical and hippocampal monoamines, reduced plasma DBH as well as elevated plasma IL-6, TNF-α, and corticosterone (CORT).

## Methods

### Animals

This study was approved by the AnimCare animal research committee (NHREC reg. no. AREC-130913-015) of the North West University (NWU) (Ethics approval number: NWU-00150-18-S5). All animals used were bred, supplied and housed at the Vivarium (SAVC reg. number FR15/13458; SANAS GLP compliance number G0019) of the Pre-Clinical Drug Development Platform (PCDDP) at the NWU.

Since FSL rats are derived from the Sprague-Dawley (SD) strain, either SD or Flinders Resistant Line (FRL) rats are used as healthy, control animals (29). Prior to beginning the study, it was essential to first establish the behavioural validity of the FSL rat with regard to depressive-like symptoms. In modelling TRD and determining the effect of FLX treatment, it was necessary to establish the presence and severity of aberrant behaviour in these animals vs. a healthy control animal, and especially vs. FSL rats reared in isolation (FSL-SIR). Comparing FSL-SIR and FSL rats to SDs gives an indication of “how close to healthy” FSL and FSL-SIR rats remit following FLX treatment. The original colonies of FSL rats were obtained from Dr David H Overstreet, University of North Carolina, USA. The effects of SIR on anxiety and hyperactivity are not consistently observed in female rats (41, 42). Since this is a requirement for the proposed model, female rats were excluded from this study. All rats were allowed free access to standard laboratory chow and water, and housed in identical transparent cages (380 mm × 380 mm × 230 mm) in an environmentally-controlled room: constant temperature (22 ± 4°C), humidity (50 ± 20%), and a 12:12 h light-dark cycle (lights on 06:00, lights off 18:00) and absence of noise. The light cycle was under white light (350–400 lux) and the dark cycle induced under red light following in-house protocol (33).

### Study Design

**Figure 1:**
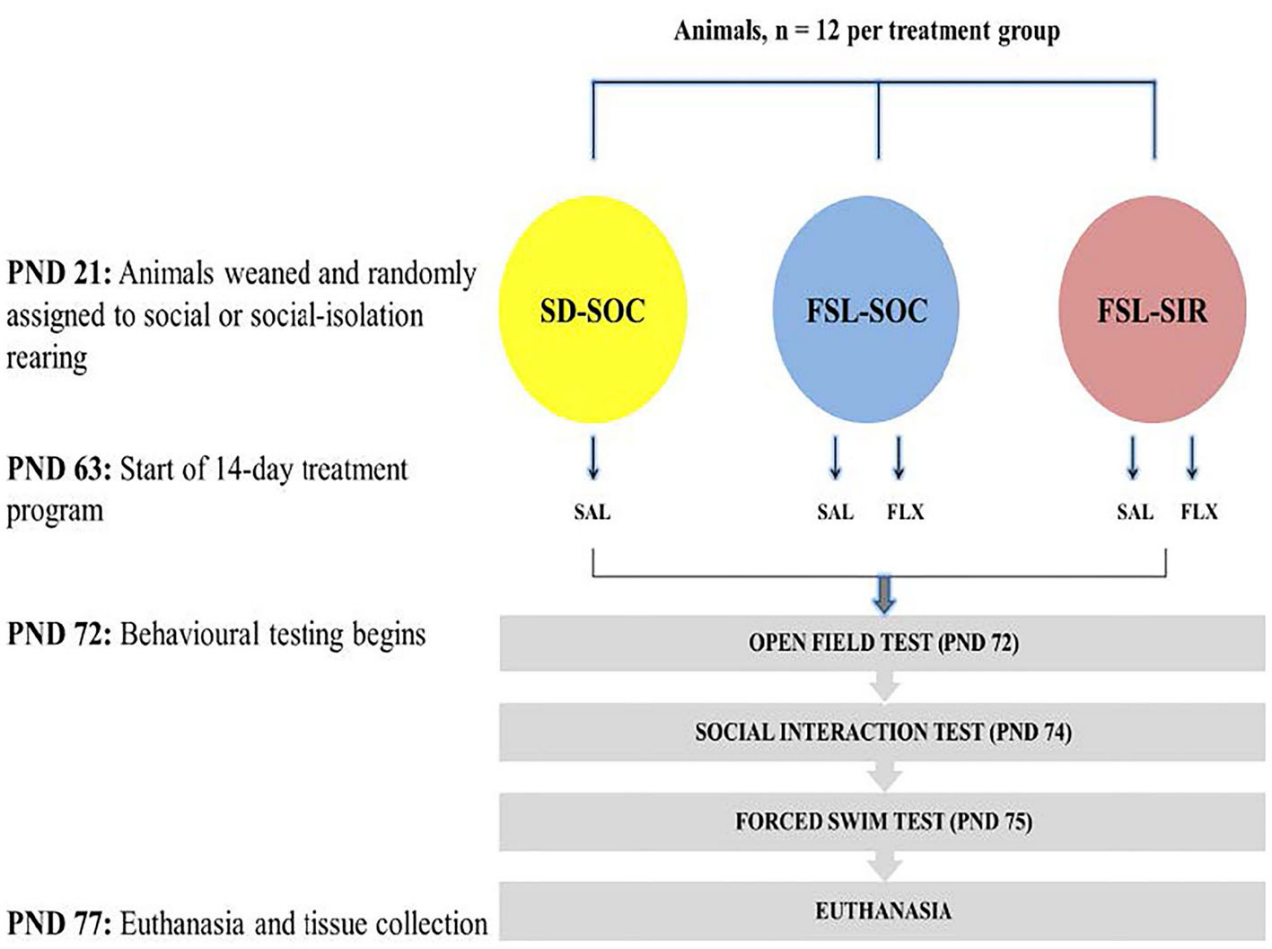
Study design.

The study design is presented in Figure 1. Animals were weaned at post-natal day (PND) 21. All SD rats were assigned to social-rearing (3 rats/cage) while FSL rats were randomly assigned to either social rearing or social isolation rearing (SIR, 1 rat/cage). Rearing conditions were maintained for a period of 8 weeks (43, 44). All animals were exposed to the same olfactory, visual, and auditory cues, although FSL-SIR rats were deprived of social contact with peer rats during this period. At PND 63, while remaining in their assigned rearing condition, FSL and FSL-SIR animals were assigned to a treatment group: either saline-treated (SAL) or FLX-treated (FLX). SD rats received only SAL. Thus, the resultant cohorts were as follows: SD-SAL, FSL-SAL, FSL-FLX, FSL-SIR-SAL, and FSL-SIR-FLX. Each cohort contained 12 rats (*n* = 12 per cohort) and a total of 60 animals were used in this study. The animals were first weighed on the day of weaning and then again each morning from the beginning of the treatment protocol (PND 63) until the last day of the study (PND 77). Their weights were used to calculate the volume of drug to be administered and to ensure equal growth in all the treatment groups. The treatment regimen commenced from PND 63 and continued until PND 76. Behavioural testing commenced on PND 72 beginning with the OFT, followed by the SIT on PND 74, and the FST on PND 75. This sequence orders the assessments from least to most stressful to ensure that the results of subsequent tests are not negatively affected by prior tests (45). All behavioural tests were performed during the dark cycle (18:30–02:30). The animals were euthanised by decapitation without prior administration of an anaesthetic 24 h after the last behavioural test. Trunk blood and brain tissue were collected for bioanalysis. For behavioural and monoamine analysis, all animals (*n* = 12 per cohort) were included in the data. For ELISA analysis, plasma samples (*n* = 10 per cohort) were randomly selected from the 12 animals per cohort. This was to allow for more samples to be assayed per plate while maintaining statistical power. Quantification of these markers in plasma as opposed to in the brain is deliberately aimed at correlating them to clinical findings which are mainly based on fluid sample readouts.

### Drug Preparation and Treatment Protocol

Fluoxetine (fluoxetine hydrochloride, or FLX; Pubchem CID 62857) was donated by Jade Pharmaceuticals, South Africa. Fluoxetine was first dissolved in approximately 500 μL distilled water and then made up to 10 mg/kg in physiological saline. Fresh FLX solution was made up daily. Fluoxetine was administered at this dose as it was shown to most reliably affect swimming behaviour (a 5-HT mediated behaviour), reduce immobility, and to robustly increase extracellular 5-HT, NE, and DA (46, 47). Fluoxetine was administered subcutaneously (s.c.) for a period of 14 days. Control rats received SAL s.c. In both instances, treatment was administered during the light cycle between 08:00 and 10:00.

### Behaviour

#### Locomotor Activity—Open Field Test

Reduced locomotor activity is a symptom of MD (48). The method of Sherif and Oreland (49) was used. Individual rats were placed into a square open field test (OFT) arena (100 × 100 × 50 cm), facing the centre of the arena. The test was conducted in a dimly lit room illuminated with red light (40 W). Animal behaviour was recorded for 5 min using a ceiling-mounted digital camera. The video files were analysed using Noldus Ethovision XT software (Noldus^®^ Information Technology, Wageningen, The Netherlands), which calculated and reported the total distance (cm) travelled within the arena.

#### Despair—Forced Swim Test

Despair is a manifest symptom of MD (29). We used a method as previously described (50). Individual rats were placed in transparent, Perspex^®^ swim tanks containing water at ambient temperature (25°C) and allowed to swim for 7 min, digitally recorded for later behavioural analysis. No pre-swim was applied as FSL rats already present with heightened immobility in the forced swim test (FST) (29). At the end of the 7-min period, the rats were removed from the cages, dried and returned to their home cages. The first and last minute of the video files were excluded from the analysis, for reasons noted earlier (51). Immobility (despair), swimming (survival, coping) and climbing (escape-driven behaviour) behaviours were scored manually by a researcher blinded to treatment and expressed as time (seconds) spent performing each behaviour. The latter are noted for representing serotonergic (swimming) and noradrenergic (climbing)-mediated escape-directed behaviour (51).

#### Social Interaction Test

Social deficits are a prominent feature of MD (48). The social interaction test/task (SIT) was performed to assess anxiety-related and social withdrawal behaviour in rodents (52–54). Behaviours are described in Table 1.

**Table 1:** Depression-related social behaviour scored in the social interaction test (SIT) [Adapted from Barnett (55), Brain et al. (56)].

**Category**	**Behaviour**	**Description**
Social	Sniffing	Sniffing the head, snout, anogenital area, or body of the partner
	Approaching	Walking directly toward the partner
	Following	Moving in close proximity to the partner as it walks around the arena
	Grooming (allo-grooming)	Grooming the body of the partner using the mouth
	Crawling over/under	Both forepaws placed on the partner, with the head and anterior part of the body pushed underneath the partner
Asocial (anxiety)	Exploring	Walking or running around the arena, not obviously directed toward the partner, supported or unsupported rearing unrelated to partner

The SIT was conducted in the same arena and under the same lighting conditions as described in the OFT. Individual animals were allowed to revisit the arena the day prior to the SIT in order to habituate to their surroundings. The familiar arena and dim lighting are conducive to maximum active behaviours (57). A pair of rats of similar mass (± 10 g) from the same treatment group and reared under the same conditions but unfamiliar to each other, were placed in the middle of the open field arena facing each other. Social and asocial behaviours were recorded using a digital camera mounted above the arena. These behaviours are strongly correlated to those evident in human MD (32). After each test session, any faecal boli were removed, urine wiped, and the arena cleaned with a 10% ethanol solution. Behaviour in the arena (Table 1) was later manually scored from videos over a 10 min observation period by an observer blind to the treatment and rearing conditions. Since the behaviour of each rat is related to its partner in the arena, pair scores (thus *n* = 6) were used (58), with each behaviour expressed as the percentage (%) time of the total duration of the session.

### Bioanalysis

#### Preparation of Plasma and Brain Tissue

Rats were decapitated, and the frontal cortex and hippocampus immediately dissected out on an ice-cooled glass slab as previously described (45, 59). Trunk blood was collected in pre-chilled, 4 mL vacutainer tubes (Vacuette^®^) containing K_3_EDTA solution as anti-coagulant. The blood was centrifuged at 1,000 × g at 4°C for 15 min. Both the brain parts and plasma were fixed in liquid nitrogen and stored at −80°C until the day of analysis.

#### Monoamine Quantification

NE, 5-HT, and DA were quantified in the hippocampus and frontal cortex using a high-performance liquid chromatography (HPLC) system with electrochemical detection (HPLC-EC), as previously described (60). The brain tissue was prepared as described by Viljoen et al. (60). Whole and regional brain monoamine analyses reflect their total extracellular and unreleased levels (61). An Agilent 1,200 series HPLC (Agilent Technologies Inc., Santa Clara, CA USA), equipped with an isocratic pump and autosampler coupled to an ESA Coulochem III Electrochemical detector with a coulometric flow cell (Model 5011A High Analytical Cell and Guard cell 5,020) and Chromeleon^®^ Chromatography Management System version 6.8 (obtained from Thermo Fisher Scientific, Waltham, MA USA), was used for this analysis.

#### Plasma Biochemistry

DBH (Catalogue no: abx256508, Abbexa, Cambridge, UK), CORT (Catalogue no: E-EL-R0269, Elabscience Biotechnology Inc., Wuhan China), IL-6 (Catalogue no: E-EL-R0015, Elabscience Biotechnology Inc., Wuhan China), and TNF-α (Catalogue no: E-EL-R0019, Elabscience Biotechnology Inc., Wuhan China) were measured by sandwich ELISA kits according to the manufacturer's protocol using a Spectronic 20 (Bausch and Lomb) spectrophotometer.

### Statistical Analysis

Statistical analysis was performed using GraphPad Prism^®^ 8 for Windows (GraphPad Software Inc., San Diego, CA, USA) under the supervision of the Statistical Consultation Service of the NWU. A power analysis was performed with a power of 80% and a significance level of 5% (*p* < 0.05) to give a minimum recommended sample size (*n* = 5) per treatment group. Previous in-house protocols have shown that sample sizes of *n* = 12 would provide sufficient tissue for the envisioned biochemical analyses (34). Repeated measures, two-way ANOVA with Bonferroni *post-hoc* test was used to analyse body weight data. Two-way ANOVA followed by Bonferroni *post-hoc* test was applied in comparisons of behaviour and biochemistry. The two-way ANOVA was selected to test the interaction between treatment and rearing condition as these were the only factors relevant to our hypothesis and aims of developing a TRD model and evaluating its response to FLX treatment. Significance was set at *p* < 0.05 for all comparisons. Where statistical significance was narrowly missed i.e., 0.05 ≤ *p* ≥ 0.06 for a parameter that would highlight specific differences between SAL- or FLX-treated FSL and FSL-SIR animals, *t*-tests and Cohen's *d* analyses were performed. Cohen's *d* value was calculated to establish the effect size and practical significance, with only large effect sizes (*d* ≥ 0.8) and very large effect sizes (*d* ≥ 1.2) (62) reported in the text and figures and discussed as required. Data are graphically presented as mean ± SEM.

## Results

### Body Weight

**Figure 2:**
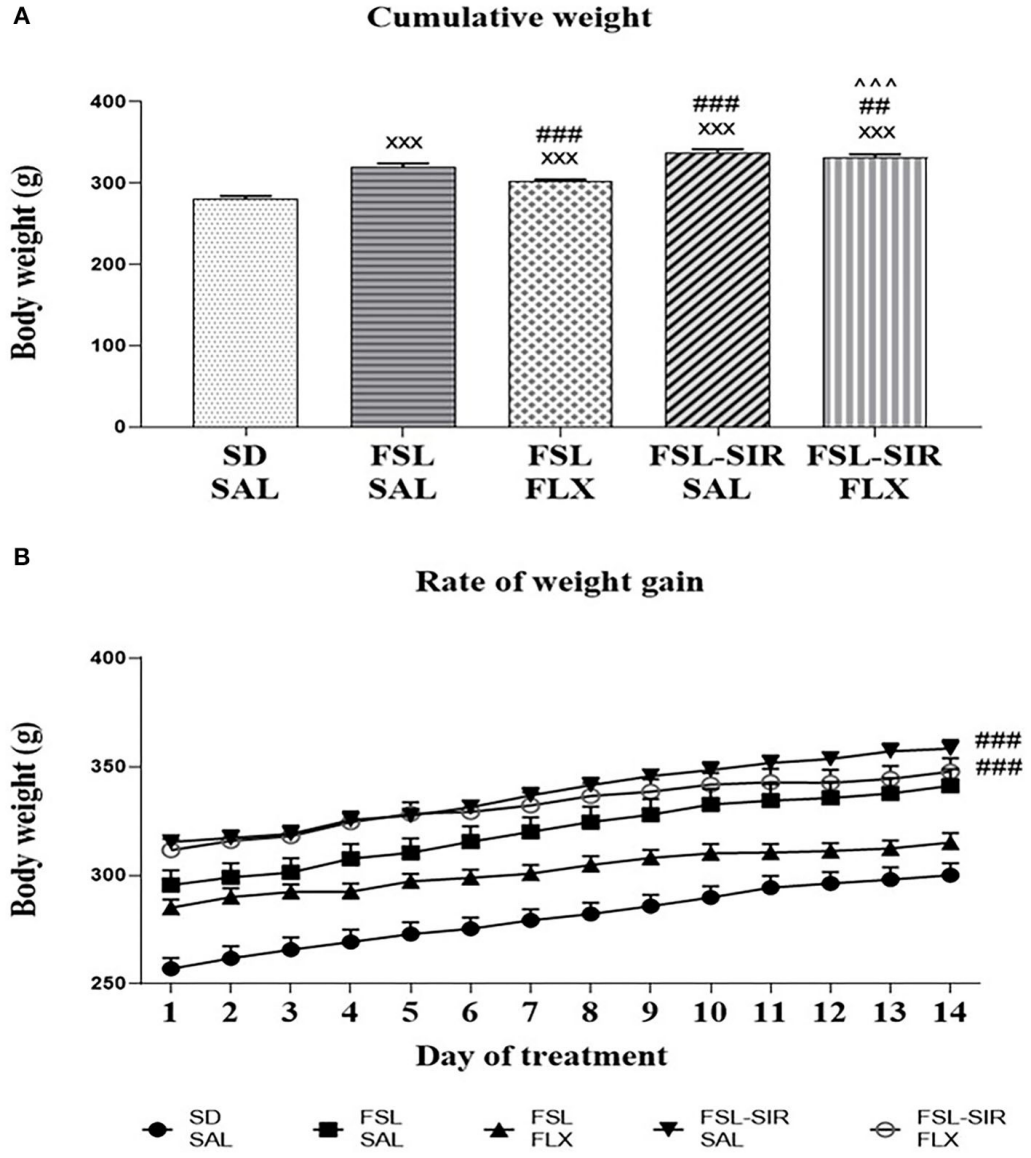
**(A)** Mean cumulative weight in SD, FSL, and FSL-SIR rats following treatment with SAL or FLX. ^xxx^*p* < 0.0001 vs. SD-SAL; ^###^*p* < 0.0001, ^##^*p* < 0.01 vs. FSL-SAL; ^∧∧∧^*p* < 0.0001 vs. FSL-FLX. **(B)** Rate of weight gain during the treatment period. ^###^*p* < 0.0001 vs. FSL-SAL. Data are represented as the mean of 12 animals. Data were analysed using two-way ANOVA followed by Bonferroni *post hoc* test. Data are presented as mean ± SEM. Precise *p*–values are presented in the text. FSL, Flinders' Sensitive Line; FLX, fluoxetine; SAL, saline; SD, Sprague-Dawley; SIR, social isolation rearing.

A significant interaction between rearing condition and treatment [*F*_(52, 715)_ = 6.769, *p* < 0.0001] as well as significant main effects of treatment [*F*_(4, 233)_ = 749.7, *p* < 0.0001] and rearing condition [*F*_(4, 55)_ = 21.51, *p* < 0.0001] were observed. As illustrated in Figure 2A, SAL-treated and FLX-treated FSL and FSL-SIR rats were all significantly heavier than SD-SAL rats (all *p* < 0.0001). While SAL-treated FSL-SIR (*p* < 0.0001) and FLX-treated FSL-SIR (*p* = 0.0001) rats were significantly heavier than SAL-treated FSL rats, FLX-treatment caused decreased weight gain compared to SAL-treatment in FSL animals (*p* < 0.0001). Fluoxetine-treated FSL-SIR rats were significantly heavier than FLX-treated FSL (*p* < 0.0001) animals. Overall rate of weight gain in SIR-exposed, SAL-treated FSL rats lagged significantly behind socially-reared, SAL-treated FSL rats (*p* < 0.0001; Figure 2B). When these groups were treated with FLX, FSL-SIR rats were observed to show significantly more rapid weight gain than FSL-SAL rats (*p* < 0.0001; Figure 2B).

### Open Field Test

**Figure 3:**
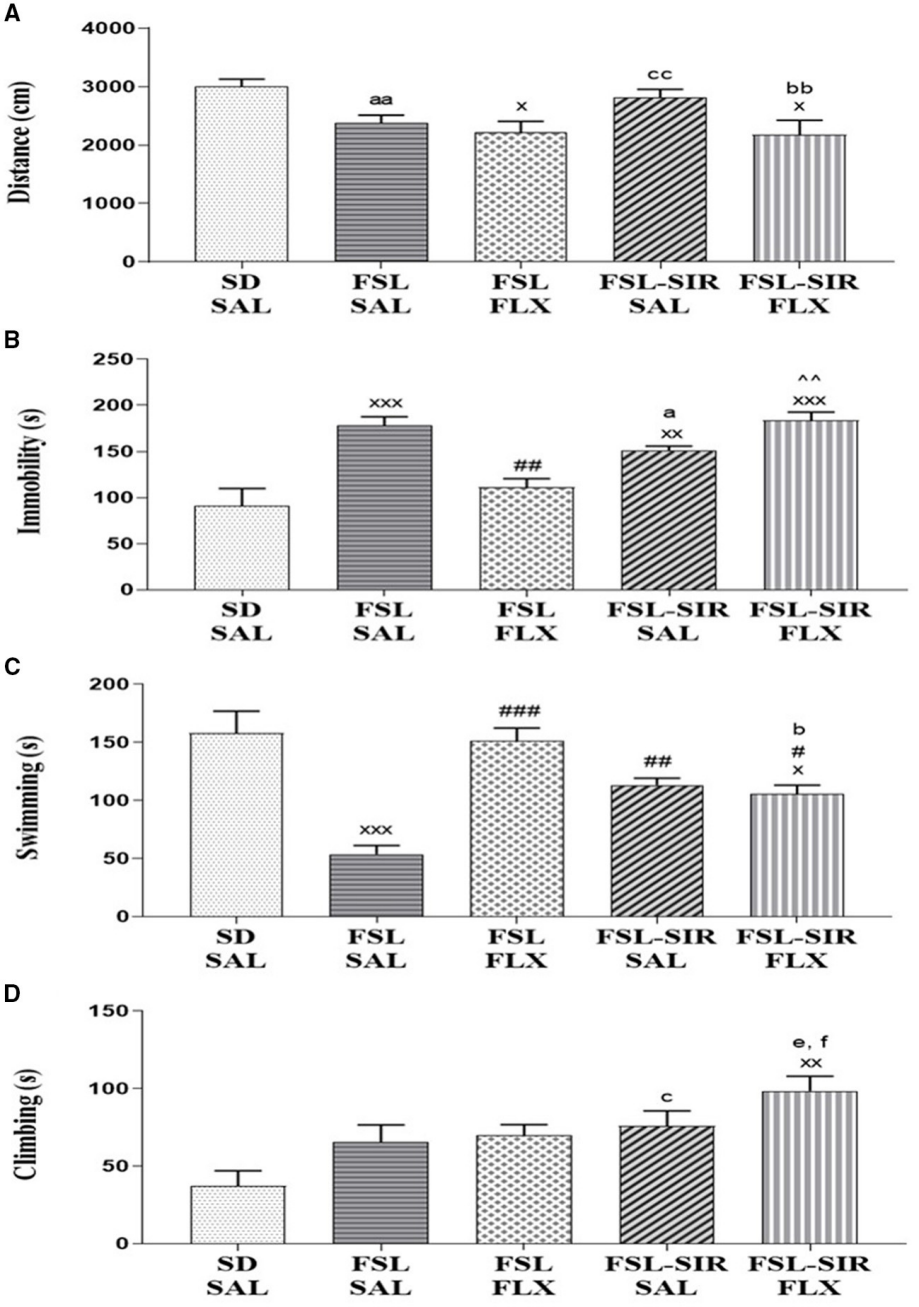
**(A)** Locomotor activity measured as distance travelled in the open field test (OFT). ^x^*p* < 0.05 vs. SD-SAL; ^aa^*d* = 1.5 vs. SD-SAL, ^cc^*d* = 0.9 vs. FSL-SAL; ^bb^*d* = 1.0 vs. FSL-SIR-SAL. **(B-D)** Immobility and swimming and climbing behaviours as measured in the forced swim test (FST). **(B)** Immobility (s). ^xxx^*p* < 0.0001, ^xx^*p* < 0.01 vs. SD-SAL; ^##^*p* < 0.01 vs. FSL-SAL; ^∧∧^*p* < 0.01 vs. FSL-FLX; ^a^*d* = 1.1 vs. FSL-SAL. **(C)** Swimming (s). ^xxx^*p* < 0.0001, ^x^*p* < 0.05 vs. SD-SAL; ^###^*p* < 0.0001, ^##^*p* < 0.01, ^#^*p* < 0.05 vs. FSL-SAL; ^b^*d* = 1.5 vs. FSL-FLX. **(D)** Climbing (s). ^xx^*p* < 0.01 vs. SD-SAL; ^c^*d* = 1.2 vs. SD-SAL, ^f^*d* = 1.0 vs. FSL-SAL, ^e^*d* = 1.0 vs. FSL-FLX. Data were analysed using two-way ANOVA followed by Bonferroni *post hoc* test and Cohen's *d* analysis. Data are presented as mean ± SEM. Detailed *p*–values in the text. FSL, Flinders' Sensitive Line; FLX, fluoxetine; SAL, saline; SD, Sprague-Dawley; SIR, social isolation rearing.

A significant main effect of rearing condition [*F*_(4, 44)_ = 4.248, *p* = 0.0054] was revealed with no main effect of treatment or rearing condition x treatment interaction observed. As illustrated in Figure 3A, FLX-treated FSL (*p* = 0.0342) and FLX-treated FSL-SIR rats (*p* = 0.0225) travelled significantly less than SD-SAL rats. Cohen's *d* analysis showed a very large effect size decrease in distance travelled by SAL-treated FSL compared to SD-SAL rats (*d* = 1.5). A large effect size decrease in locomotor activity was observed in FLX-treated FSL-SIR compared to SAL-treated FSL-SIR rats (*d* = 1.0), while a large effect size increase in activity was observed in SAL-treated FSL-SIR compared to FSL-SAL rats (*d* = 0.9).

### Forced Swim Test

*Immobility* (Figure 3B). A significant main effect of rearing condition [*F*_(4, 44)_ = 12.24, *p* < 0.0001] but no rearing condition x treatment interaction or main effect of treatment was observed. Significantly increased immobility was observed in FSL-SAL (*p* < 0.0001), FSL-SIR-SAL (*p* = 0.0078), and FSL-SIR-FLX (*p* < 0.0001) compared to SD-SAL rats. Fluoxetine–treatment significantly decreased immobility in the FSL rats compared to FSL-SAL (*p* = 0.0019). Fluoxetine-treatment significantly raised immobility in FSL-SIR compared to FLX-treated FSL rats (*p* = 0.0007). Cohen's *d* showed a large effect size decrease in immobility in FSL-SIR-SAL compared to FSL-SAL rats (*d* = 1.1).

*Swimming* (Figure 3C). A significant main effect of rearing condition [*F*_(4, 44)_ = 13.34, *p* < 0.0001] was indicated, although there was no rearing condition x treatment interaction or main effect of treatment. SAL-treated FSL (*p* < 0.0001) and FLX-treated FSL-SIR rats (*p* = 0.0229) spent significantly less time swimming compared to SD-SAL rats. A significant increase in swimming was observed in FSL-SIR-SAL (*p* = 0.0064), FSL-FLX (*p* < 0.0001) and FSL-SIR-FLX (*p* = 0.0262) rats compared to SAL-treated FSL rats. Cohen's *d* showed a very large effect size decrease in swimming in FLX-treated FSL-SIR compared to FLX-treated FSL rats (*d* = 1.5).

*Climbing* (Figure 3D). A significant main effect of rearing condition [*F*_(4, 44)_ = 5.269, *p* = 0.0015] was noted, although there was no rearing condition x treatment interaction or main effect of treatment. Fluoxetine treatment significantly increased climbing in FSL-SIR compared to SD-SAL rats (*p* = 0.0005). Cohen's *d* showed a large effect size increase in climbing in SAL-treated FSL-SIR compared to SD-SAL rats (*d* = 1.2). Similarly, a large effect size increase in climbing in FLX-treated FSL-SIR compared to FSL-FLX (*d* = 1.0) and FSL-SAL rats (*d* = 1.0) was observed.

### Social Interaction Test

**Figure 4:**
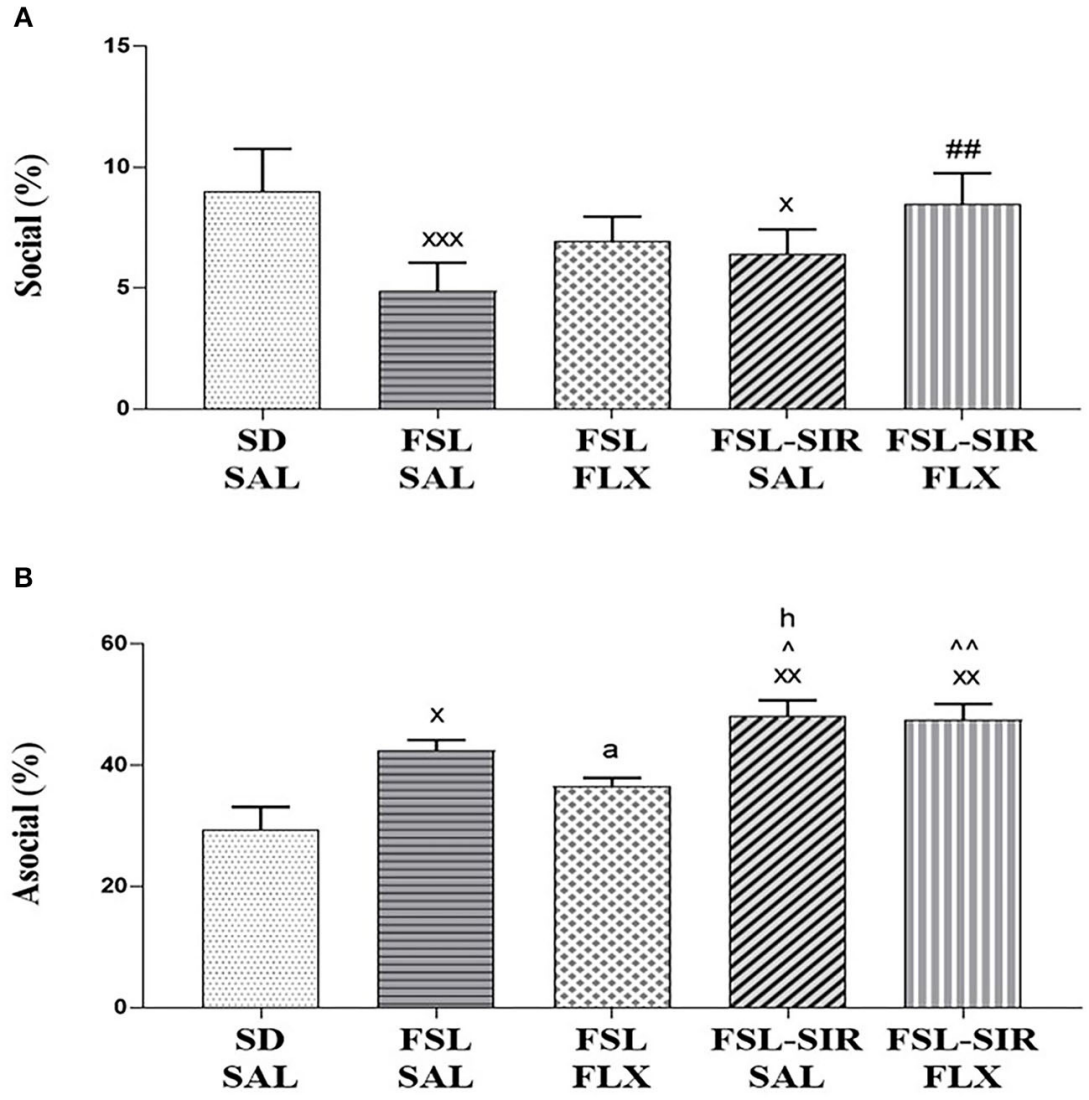
Social interactive behaviour as measured in the social interaction test (SIT). **(A)** Social (amicable) behaviour. ^xxx^*p* < 0.0001, ^x^*p* < 0.05 vs. SD-SAL; ^##^*p* < 0.01 vs. FSL-SAL. **(B)** Asocial/socially anxious-like behaviour. ^xx^*p* < 0.01, ^x^*p* < 0.05 vs. SD-SAL; ^∧∧^*p* < 0.01, ^∧^*p* < 0.05 vs. FSL-FLX; ^a^*d* = 1.7, ^h^*d* = 1.1 vs. FSL-SAL. Data were analysed using two-way ANOVA followed by Bonferroni *post hoc* test and Cohen's *d* analysis and Student's *t*-test with Welch's correction. Data are presented as mean ± SEM. Detailed *p*–values in the text. FSL, Flinders' Sensitive Line; FLX, fluoxetine; SAL, saline; SD, Sprague-Dawley; SIR, social isolation rearing.

*Social* (amicable; Figure 4A). A significant main effect of treatment [*F*_(29, 116)_ = 17.85, *p* < 0.0001] and of rearing condition [*F*_(4, 116)_ = 7.317, *p* < 0.0001] was revealed, although there was no treatment x rearing condition interaction. A significant decrease in social behaviour was observed in FSL-SAL (*p* < 0.0001) and FSL-SIR-SAL (*p* = 0.0304) rats compared to SD-SAL rats. Fluoxetine treatment significantly increased social behaviour in FSL-SIR rats compared to SAL-treated FSL rats (*p* = 0.0007).

*Asocial* (social anxiety; Figure 4B). A significant main effect of rearing condition [*F*_(4, 20)_ = 10.07, *p* = 0.0001] was observed without significant treatment x rearing condition interaction, and no main effect of treatment was noted. A significant increase in asocial behaviour was observed in FSL-SAL (*p* = 0.0139), FSL-SIR-SAL (*p* = 0.0003), and FSL-SIR-FLX (*p* = 0.0005) groups compared to SD-SAL rats. FSL-SIR-SAL rats exhibited significantly more asocial behaviour compared to FSL-FLX rats (*p* = 0.0388). FSL-SIR-FLX narrowly missed significance in a two-way ANOVA compared to FSL-FLX rats (*p* = 0.0542). To validate the treatment-resistant nature of FSL-SIR rats to FLX, a *t*-test revealed a significant increase in asocial behaviour in FSL-SIR-FLX compared to FSL-FLX rats (*p* = 0.0066). The data for the *t*-test was checked for normality using Shapiro-Wilk's test and found to be normally distributed. Cohen's *d* analysis revealed a large effect size increase in asocial behaviour in FSL-SIR-SAL compared to FSL-SAL rats (*d* = 1.1). A very large effect size reduction of asocial behaviour was noted in FSL rats following FLX treatment (*d* = 1.7).

### Monoamines

**Figure 5:**
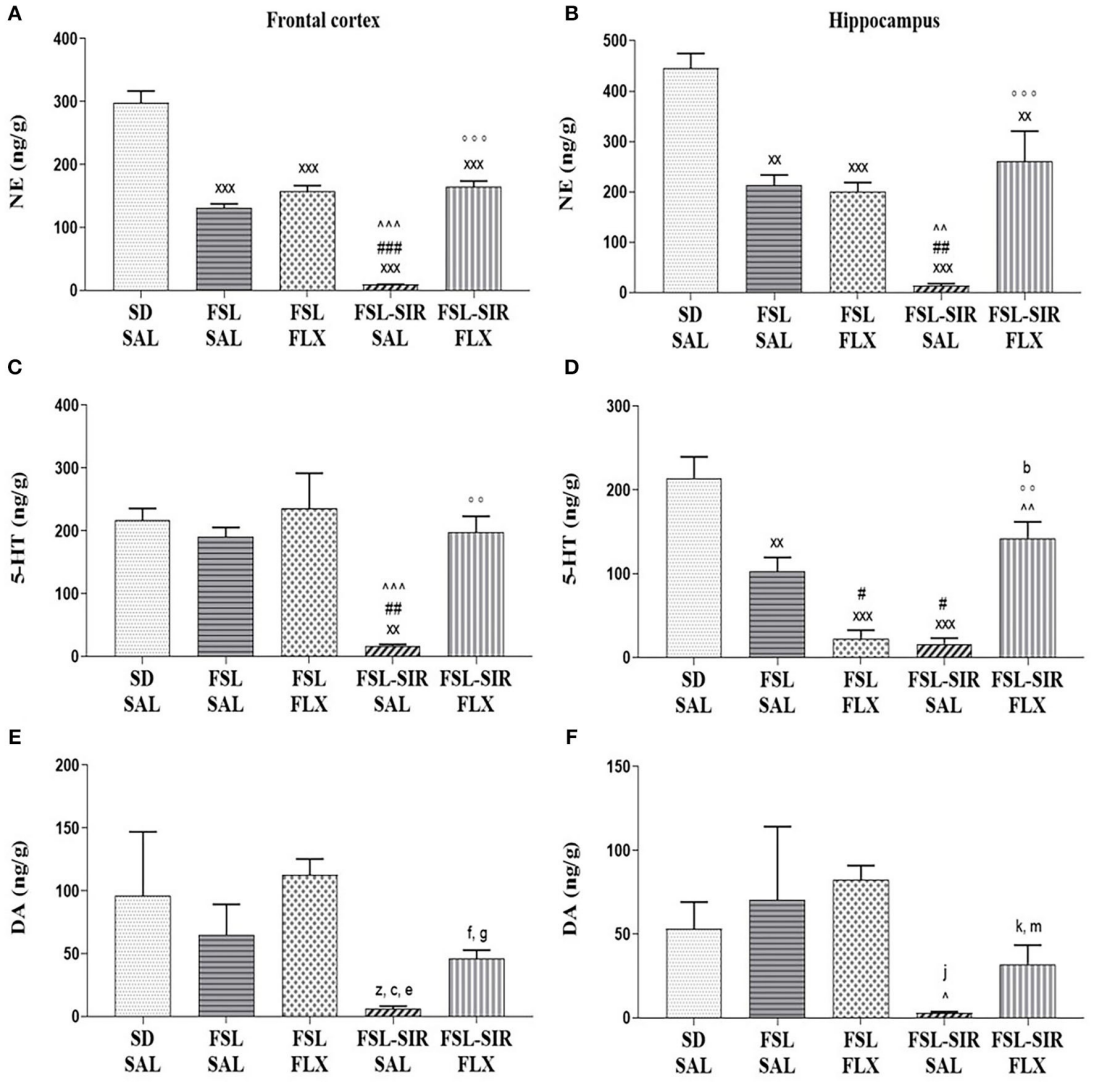
Monoamine levels in the frontal cortex **(A,C,E)** and hippocampus **(B,D,F)**. **(A)** NE. ^xxx^*p* < 0.0001 vs. SD-SAL; ^###^*p* < 0.0001 vs. FSL-SAL; ^∧∧∧^*p* < 0.0001 vs. FSL-FLX; ^°°°^*p* < 0.0001 vs. FSL-SIR-SAL. **(B)** NE. ^xxx^*p* < 0.0001, ^xx^*p* < 0.01 vs. SD-SAL; ^##^*p* < 0.01 vs. FSL-SAL; ^∧∧^*p* < 0.01 vs. FSL-FLX; ^°°°^*p* < 0.0001 vs. FSL-SIR-SAL. **(C)** 5-HT. ^xx^*p* < 0.01 vs. SD-SAL; ^##^*p* < 0.01 vs. FSL-SAL; ^∧∧∧^*p* < 0.0001 vs. FSL-FLX; ^°°^*p* < 0.01 vs. FSL-SIR-SAL. **(D)** 5-HT. ^xxx^*p* < 0.0001, ^xx^*p* < 0.01 vs. SD-SAL; ^#^*p* < 0.05 vs. FSL-SAL; ^∧∧^*p* < 0.01 vs. FSL-FLX, ^°°^*p* < 0.01 vs. FSL-SIR-SAL. ^b^*d* = 1.0 vs. SD-SAL. **(E)** DA. ^z^*d* = 0.8 vs. SD-SAL; ^c^*d* = 1.0 vs. FSL-SAL, ^e^*d* = 3.5, ^f^*d* = 2.0 vs. FSL-FLX, ^g^*d* = 2.4 vs. FSL-SIR-SAL. **(F)** DA. ^∧^*p* < 0.05 vs. FSL-FLX. ^j^*d* = 1.3 vs. SD-SAL, ^k^*d* = 1.5 vs. FSL-FLX; ^m^*d* = 1.1 vs. FSL-SIR-SAL. Data were analysed using two-way ANOVA followed by Bonferroni *post hoc* test and Cohen's *d* analysis. Data are presented as mean ± SEM. Detailed *p*–values in the text. DA, dopamine; FSL, Flinders' Sensitive Line; FLX, fluoxetine; NE, norepinephrine; SAL, saline; 5-HT, serotonin; SIR, social isolation rearing; SD, Sprague-Dawley.

*NE: Frontal cortex* (Figure 5A). A significant main effect of rearing condition [*F*_(4, 44)_ = 84.57, *p* < 0.0001], but with no main effect of treatment and no treatment x rearing condition interaction was evident. Significantly reduced levels of frontocortical NE were observed in FSL-SAL, FSL-FLX, FSL-SIR-SAL, and FSL-SIR-FLX rats compared to SD-SAL rats (all *p* < 0.0001). FSL-SIR-SAL rats were found to have significantly diminished NE levels in this brain region compared to FSL-SAL (*p* < 0.0001) and FSL-FLX rats (*p* < 0.0001). A significant increase in frontocortical NE was observed in the FLX-treated FSL-SIR rats compared to SAL-treated FSL-SIR rats (*p* < 0.0001).

*Hippocampus* (Figure 5B). A significant main effect of rearing condition [*F*_(4, 44)_ = 20.08, *p* < 0.0001] was observed, although no treatment x rearing condition interaction or main effect of treatment was observed. Significantly reduced hippocampal NE levels were observed in FSL-SAL (*p* = 0.0002), FSL-FLX (*p* < 0.0001), FSL-SIR-SAL (*p* < 0.0001), and FSL-SIR-FLX (*p* = 0.0046) groups compared to SD-SAL rats. There were significantly diminished hippocampal NE levels in SAL-treated FSL-SIR compared to FSL-SAL (*p* = 0.0018) and FSL-FLX (*p* = 0.0036) rats. Fluoxetine treatment significantly increased hippocampal NE in FSL-SIR rats compared to SAL-treatment (*p* < 0.0001).

*5-HT: Frontal cortex* (Figure 5C). A significant main effect of rearing condition [*F*_(4, 44)_ = 21.63, *p* < 0.0001] was noted, although no treatment x rearing condition interaction or main effect of treatment was evident. SAL-treated FSL-SIR rats presented with significantly reduced frontocortical 5-HT compared to SD-SAL (*p* = 0.0002), FSL-SAL (*p* = 0.0013), and FSL-FLX rats (*p* < 0.0001). Fluoxetine-treated FSL-SIR rats had significantly elevated 5-HT levels in this region compared to SAL-treated FSL-SIR rats (*p* = 0.0007).

*Hippocampus* (Figure 5D). A significant main effect of rearing condition [*F*_(4, 44)_ = 21.63, *p* < 0.0001] was noted, although there was no treatment x rearing condition interaction or main effect of treatment. SAL-treated FSL (*p* = 0.0007), FSL-FLX and FSL-SIR-SAL rats (both *p* < 0.0001) presented with significantly reduced 5-HT compared to SD-SAL rats. Significantly diminished hippocampal 5-HT was observed in FLX-treated FSL (*p* = 0.0311) and SAL-treated FSL-SIR (*p* = 0.0152) rats compared to SAL-treated FSL rats. FLX significantly raised 5-HT in FSL-SIR rats compared to FLX-treated FSL (*p* = 0.0003) and SAL-treated FSL-SIR rats (*p* = 0.0001). Cohen's *d* analysis showed a large effect size reduction in hippocampal 5-HT in FLX-treated FSL-SIR rats compared to SD-SAL controls (*d* = 1.0).

*DA: Frontal cortex* (Figure 5E). Cohen's *d* analysis showed a large effect size reduction in frontocortical DA in SAL-treated FSL-SIR compared to SD-SAL (*d* = 0.8) and SAL-treated FSL rats (*d* = 1.1), and a very large effect size reduction compared to FLX-treated FSL rats (*d* = 3.5). Fluoxetine treatment caused a very large effect size increase in frontocortical DA in FSL-SIR compared to FSL-FLX rats (*d* = 2.0) and a very large elevation compared to SAL-treated FSL-SIR rats (*d* = 2.4).

*Hippocampus* (Figure 5F). A significant effect of treatment [*F*_(11, 44)_ = 2.576, *p* = 0.0129] and of rearing condition [*F*_(4, 44)_ = 2.784, *p* = 0.0380], but without interaction between the two factors, was observed. Hippocampal DA was significantly reduced in SAL-treated FSL-SIR compared to FLX-treated FSL rats (*p* = 0.0490). Cohen's *d* analysis showed a very large effect size reduction in DA in SAL-treated FSL-SIR rats compared to SD-SAL rats (*d* = 1.3). A large effect size reduction was also observed in FLX-treated FSL-SIR compared to FLX-treated FSL rats (*d* = 1.5). Fluoxetine caused a large effect size increase in FSL-SIR compared to SAL-treated FSL-SIR rats (*d* = 1.1).

### Plasma Biochemistry

**Figure 6:**
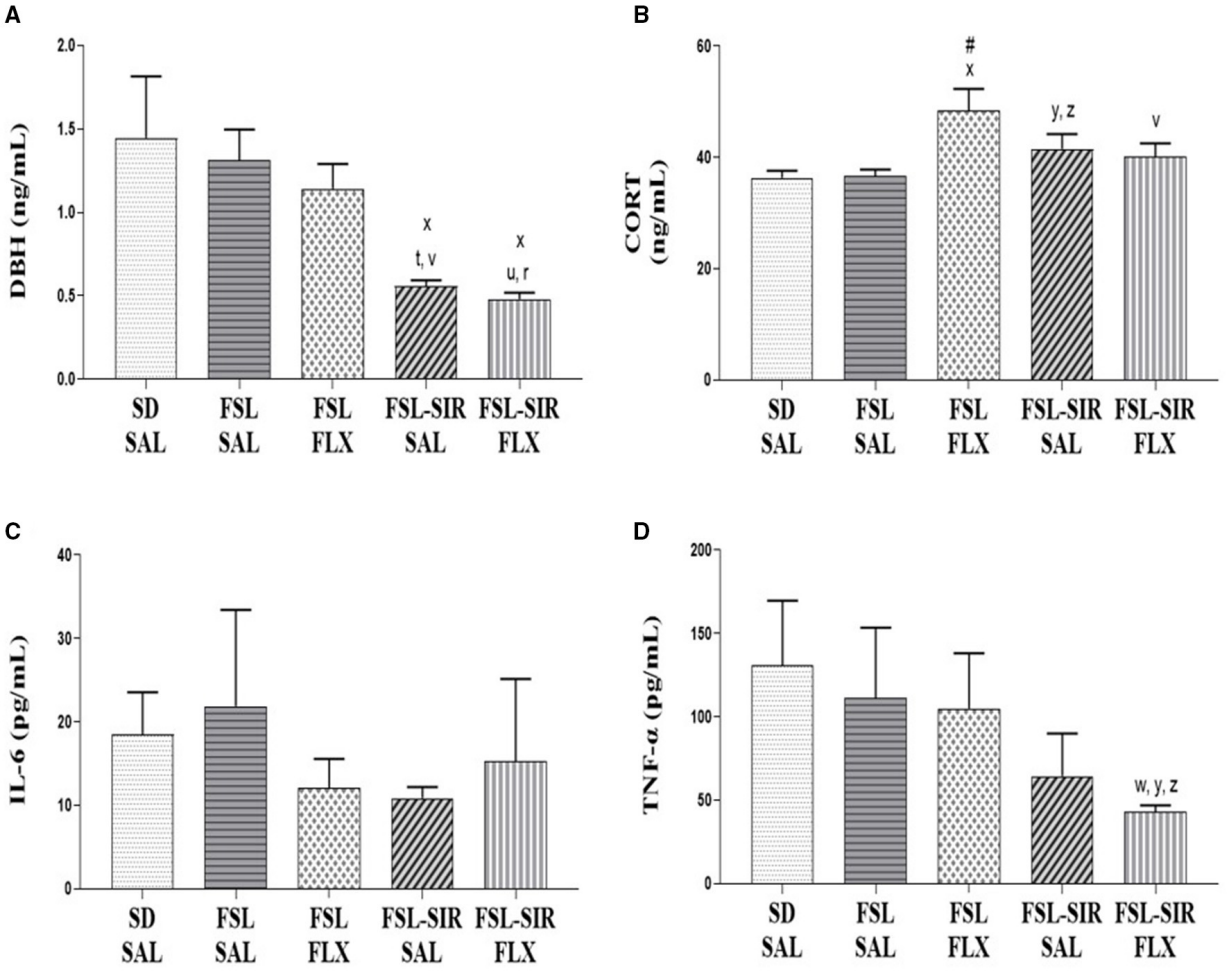
Plasma biochemistry. **(A)** DBH. ^x^*p* < 0.05 vs. SD-SAL; ^t^*d* = 1.9, ^r^*d* = 2.1 vs. FSL-SAL; ^v^*d* = 1.8, ^u^*d* = 2.0 vs. FSL-FLX. **(B)** CORT. ^x^*p* < 0.05 vs. SD-SAL; ^#^*p* < 0.05 vs. FSL-SAL; ^y^*d* = 0.8 vs. SD-SAL; ^z^*d* = 0.8 vs. FSL-SAL; ^v^*d* = 0.8 vs. FSL-FLX. **(C)** IL-6. No significant effects between cohorts were observed. **(D)** TNF-α. ^y^*d* = 1.1 vs. SD-SAL; ^w^*d* = 0.8 vs. FSL-SAL; ^z^*d* = 0.9 vs. FSL-FLX. Data were analysed using two-way ANOVA followed by Bonferroni *post hoc* test and Cohen's *d* analysis. Data are presented as mean ± SEM. Detailed *p*–values in the text. CORT, corticosterone; DBH, dopamine-beta-hydroxylase; FSL, Flinders' Sensitive Line; FLX, fluoxetine; IL-6, interleukin 6; SAL, saline; SD, Sprague-Dawley; SIR, social isolation rearing; TNF-α, tumour necrosis factor-alpha.

*DBH* (Figure 6A). A significant main effect of rearing condition [*F*_(4, 36)_ = 4.841, *p* = 0.0032] was noted, with no main effect of treatment and no interaction between these two factors observed. SAL-treated FSL-SIR (*p* = 0.0350) and FLX-treated FSL-SIR (*p* = 0.0166) rats presented with significantly reduced levels of plasma DBH compared to SD-SAL rats. Cohen's *d* showed very large effect size reductions in plasma DBH in SAL-treated FSL-SIR compared to SAL-treated FSL (*d* = 1.9) and FSL-FLX rats (*d* = 1.8), and in FLX-treated FSL-SIR compared to SAL-treated FSL (*d* = 2.1) and FLX-treated FSL rats (*d* = 2.0).

*CORT* (Figure 6B). A significant main effect of rearing condition [*F*_(4, 36)_ = 3.412, *p* = 0.0183] was indicated, but without a main effect of treatment or treatment x rearing condition interaction. Fluoxetine-treated FSL rats presented with significantly elevated plasma CORT levels compared to SD-SAL (*p* = 0.0276) and SAL-treated FSL rats (*p* = 0.0335). SIR-FSL rats increased CORT levels vs. SD-SAL and FSL-SAL rats by a large effect size (both *d* = 0.8). A large effect size reduction in plasma CORT was evident in FLX-treated FSL-SIR compared to FLX-treated FSL rats (*d* = 0.8), as revealed by Cohen's *d* analysis.

*IL-6* (Figure 6C). No significant differences between cohorts were found.

*TNF-*α (Figure 6D). Cohen's *d* showed a large effect size reduction of plasma TNF-α in FSL-SIR-FLX compared to SD-SAL (*d* = 1.0), SAL-treated FSL (*d* = 0.8), and FLX-treated FSL rats (*d* = 0.9).

## Discussion

Key findings are that FSL rats exposed to SIR presented with depressive-like symptoms manifesting as reduced swimming and prolonged immobility in the FST similar to socially-reared FSL rats. However, these behaviours *worsened* in response to FLX, while worse social impairment in FSL-SIR rats also showed non-response to FLX. Biochemically, FSL-SIR rats presented with diminished DA, NE and 5-HT in both the frontal cortex and hippocampus, which although raised by FLX, mostly remained suppressed compared to the healthy control (SD-SAL), as well as had reduced plasma DBH which was unaffected by FLX. Together these data indicate treatment resistance to a first-line antidepressant.

Reduced body weight of FSL rats is well-described (30), with FSL and FSL-SIR rats gaining weight more slowly than SD-SAL rats (Figure 2B), congruent with similar animal models (63). Compromised weight gain is typical of chronic stress, such as SIR (64), as evident in SAL-treated FSL-SIR rats. Interestingly, patients with TRD have a higher BMI and tend to be obese (12, 65). Fluoxetine treatment reduced weight gain in social and isolated FSL rats, in line with its anorexigenic effects (66), although like in patients with TRD (12), FLX-treated FSL-SIR rats gained weight significantly faster than SAL-treated FSL rats.

Consistent with literature (67), FSL rats displayed psychomotor retardation in the OFT compared to SD-SAL rats (*d* = 1.5, Figure 3A), with no significant locomotor effects evident in FSL-SIR vs. SD-SAL rats (Figure 3A). However, consistent with TRD vs. MD patients (23, 68), locomotor activity was increased in SAL-treated FSL-SIR rats vs. FSL-SAL rats (Figure 3A), which in turn was significantly decreased by FLX in FSL-SIR rats (Figure 3A).

FSL rats displayed significantly elevated immobility (despair) and reduced swimming (survival behaviour) in the FST vs. SD-SAL rats as well as being reversed by FLX (Figures 3B,C), indicative of a 5-HT-selective antidepressant. FSL-SIR rats also displayed marked immobility and reduced swimming vs. SD-SAL animals, although immobility was less than in FSL-SAL animals (*d* = 1.1). However, the antidepressant effects of FLX were negated in FSL-SIR rats, with significantly increased immobility and decreased swimming vs. SD-SAL rats (Figures 3B,C). Climbing behaviour was largely unaffected by rearing condition or treatment in FSL-SAL rats (Figure 3D), but was raised by SIR and FLX in FSL-SIR rats which argues *against* increased immobility being a confounding variable in the FST in FLX-treated FSL-SIR rats.

FSL-SIR-SAL rats displayed more swimming than FSL-SAL rats. This increase in swimming behaviour has been observed in other strains of rats reared in isolation (69). Since the OFT (Figure 3A), SIT (Figure 4B, later in the discussion) and immobility (Figure 3B) data indicate abnormal (depressive-like) behaviour in this animal, our interpretation is that SIR may invoke a reactive response to a stressful situation (FST) in FSL rats manifesting as increased swimming compared to their socially-reared counterparts. SIR also increased climbing behaviour in the FSL rats. While climbing behaviour typically suggests increased escape-driven behaviour, it has also been associated with anxious behaviour (70).

FLX treatment increased immobility (Figure 3B) and climbing (Figure 3D) behaviour in FSL-SIR rats but left swimming (Figure 3B) relatively unchanged compared to SAL treatment in the same rats. This exacerbation of depressive- (swimming) and anxiety-like (climbing) behaviour could speak to the bidirectional role of 5HT_1A_ receptors in the response to stress, where elevated 5-HT has been suggested to have opposing actions in mood and anxiety disorders (71, 72). Worsening of depression including worsening of agitation (anxiety) is considered a form of treatment resistance that in the clinical environment may be related to increased suicidal behaviour, that in turn may be linked to psychotic features presenting in MD (73) or bipolar diathesis (74). Considering that post-weaning isolation reared rats typically present with psychotic-like symptoms, it is possible that FSL-SIR rats show abnormal/paradoxical response to FLX-treatment due to untreated psychotic features. This warrants further study.

Consistent with literature (67, 75), FSL rats displayed social withdrawal (Figure 4A), a key symptom of MD (76), and increased arena exploration in the SIT (Figure 4B), the latter a measure of anxiety (58, 77). Anxiety and depression are notoriously comorbid (78), and although not a core trait of the FSL rat (29), anxiety has been noted in the SIT (67) as re-affirmed here (Figure 4B). Moreover, and congruent with literature (79, 80), these anxiety-like behaviours were effectively reversed by FLX (*d* = 1.7, Figure 4B). Social deprivation decreases social interactive behaviour and increases anxiety in adulthood (33). Not surprising then that FSL-SIR animals exhibited greater social anxiety-like behaviour than the FSL group (*d* = 1.1). Similarly, FSL-SIR rats exhibited disordered social behaviour and asocial (anxious) behaviour vs. SD-SAL rats (Figures 4A,B). Although SSRIs are anxiolytic in FSL rats (79, 80), see also Figure 4B, FLX showed no anxiolytic effect in FSL-SIR rats (Figure 4B), which alludes to an underlying neurobiological change following SIR that prompts resistance to the anxiolytic effects of chronic FLX treatment. Furthermore, this non-response to FLX is also supportive of the increased climbing behaviour in the FST indicating an animal with increased anxiety/agitation; congruent with clinical TRD (74).

The monoamine hypothesis suggests that depressive symptoms result from deficits in NE and 5-HT neurotransmission (28, 81), a resulting upregulation of 5-HT receptors (5-HT_1A_, _2A, 2C, 4_) (82–84), and up-regulation and increased sensitivity of NE receptors (particularly α_2_ adrenergic) (28, 85). FSL rats present with a blunted 5-HT response (67), reduced 5-HT transporters (SERT) (86) as well as elevated cortico-hippocampal 5-HT (29, 87). Here, frontocortical 5-HT in the FSL rats was unperturbed. FSL rats displayed reduced 5-HT in the hippocampus (Figure 5D), coinciding with reduced swimming (Figure 3B), in agreement with the biogenic amine theory of MD. On the other hand, juvenile adversity (e.g., SIR) reduces the density and attenuates function of post-synaptic 5-HT_1A_ receptors in the hippocampus and stress centres of the brain (88–90) and reduces presynaptic serotonergic function (89). This would explain the significantly reduced cortico-hippocampal 5-HT levels in FSL-SIR vs. SAL-treated FSL rats (Figures 5C,D), with reduced swimming (Figure 3B). That said, although depletion of 5-HT has been shown to block the action of SSRIs (91), FSL-SIR rats treated with FLX still presented with significantly increased cortico-hippocampal 5-HT (Figures 5C,D), and increased swimming (Figure 3B), yet now displayed *exacerbated* depressive behaviours (immobility; Figure 3B). This could speak to the bidirectional role of 5HT_1A_ receptors in the response to stress (72), where elevated 5-HT has been suggested to have opposing actions on mood (71).

FSL rats also presented with reduced NE in the frontal cortex and hippocampus vs. SD-SAL controls (Figures 5A,B) correlating with the monoaminergic theory of MD. However, FLX did not alter this, nor was there an effect on climbing behaviour (Figure 3D), thus excluding a possible noradrenergic mode of action (92). Similarly, FSL-SIR-SAL animals presented with reduced cortico-hippocampal NE vs. both SD-SAL and SAL-treated FSL groups, although levels were further and significantly reduced in FSL-SIR rats vs. FSL-FLX rats (Figures 5A,B). Hyponoradrenergia is associated with social withdrawal (39), supportive of the social impairment seen in FSL and FSL-SIR rats (Figures 4A,B). Psychosocial impairments are also highly prevalent in TRD (93) and correlate with low cortico-hippocampal NE levels in FSL-SIR rats. Fluoxetine aided in the recovery of NE in FSL-SIR rats, supportive of the increased climbing of FSL-SIR animals in the FST (Figure 3D), although with only slight improvement in social behaviour in FSL-SIR vs. FSL-SAL rats.

FSL rats presented with unaltered cortico-hippocampal DA levels vs. SD-SAL controls (Figures 5E,F), corresponding with previous findings (94). However, frontocortical (*d* = 0.8) and hippocampal (*d* = 1.3) DA levels were reduced in FSL-SIR vs. SD-SAL rats (Figures 5E,F), congruent with the monoaminergic theory of depression (28), as well as being evident in patients with TRD (21, 22). Fluoxetine increases cortical and hippocampal DA (47, 95), also evident in FSL-SIR vs. FSL-SIR-SAL rats (Figures 5E,F). These data are intriguing and warrant further study using a model that co-presents with depression and psychosis-like behaviour, with possible relevance for bipolar disorder.

As in MD literature (96), plasma DBH levels were unchanged in FSL vs. SD-SAL rats (Figure 6A), also unaffected by FLX treatment. However, DBH was significantly reduced in FSL-SIR-SAL vs. SD-SAL rats, with a very large effect size reduction vs. SAL-treated FSL rats, not unlike that described in the clinical literature (97, 98). With reduced DBH being a putative biomarker of TRD (31), these findings confirm our earlier monoamine data, especially DA, as well as behavioural data supporting a TRD animal model. While FLX treatment elevated cortico-hippocampal DA levels in FSL rats (Figures 5E,F), it did not change DBH (Figure 6A), as noted elsewhere (98). Elevated DA could therefore be a direct consequence of insufficient DBH conversion from NE. The inability of FLX to reverse lowered DBH levels in FSL-SIR animals suggests a SIR-induced change to FSL neurobiology that resists response to FLX, and warrants further validation using an atypical antipsychotic.

FSL rats presented with unaltered basal plasma CORT levels vs. SD-SAL rats (Figure 6B), not unusual in this model (99). TRD is associated with elevated CORT (24), supported by findings in the FSL-SIR model (Figure 6B). Interestingly, FLX treatment exacerbated the CORT response in FSL rats (Figure 6B). Fluoxetine treatment of ≤ 2 weeks may have a stimulatory effect on the rodent HPA-axis (100), possibly by up-regulating glucocorticoid receptors (101). Indeed, sub-chronic (9 days) FLX treatment has been found to down-regulate glucocorticoid receptor expression and to increase CORT (100). Unlike in FSL-FLX rats, CORT was unaffected by FLX treatment in FSL-SIR rats (Figure 6B).

Elevated IL-6 and TNF-α have been causally linked to MD (28), while elevated IL-1α has been described in FSL rats (102). Here, neither plasma IL-6 nor TNF-α were altered in FSL vs. SD-SAL rats, and were unaffected by FLX (Figures 6C,D). Although elevations in TNF-α and IL-6 are described in MD, their role in predicting antidepressant resistance is debated (103). However, while FLX had no effect on IL-6 in FSL or FSL-SIR rats, it engendered a large effect size decrease in TNF-α in FSL-SIR vs. SD-SAL and FLX-treated FSL rats (Figure 6D). Fluoxetine has anti-inflammatory effects via serotonergic transmission and activation of the HPA-axis (104, 105), which we also observed in FSL rats (Figure 6B). That FSL-SIR rats did not exhibit significant elevations in these cytokines or plasma CORT may point toward inherent biological mechanisms that protect against severe combined insults. This is especially evident in dual-hit models (106). In this regard, an adverse early-life experience is said to trigger an adaptive process that renders an individual better adapted or resilient to stressful environments later in life (40, 107, 108).

In summary (Table 2), FSL-SIR rats were significantly heavier than FSL rats in response to treatment, a symptom of TRD (12), and where weight loss is typically seen following FLX-treatment, this was not demonstrated in FSL-SIR rats. FSL-SIR rats also showed a large effect size increase in locomotor activity vs. FSL rats, consistent with TRD literature (23, 109, 110). TRD presents with exaggerated symptoms of MD in addition to antidepressant resistance (109, 111). FSL-SIR rats presented with reduced coping behaviours, increased depressive-like behaviour, and increased social withdrawal and social anxiety, together with altered cortico-hippocampal NE and 5-HT levels supportive of deficits in coping and escape-directed strategies. Although FSL-SIR rats showed similar behaviour deficits to FSL rats, they were not more severe. Importantly, while biogenic amine anomalies were variably improved by FLX, social deficits remained unresponsive while depressive symptoms worsened following FLX treatment. In addition, FSL-SIR rats demonstrated a large effect size increase in CORT and a very large effect size reduction in DBH levels, recognised biomarkers of TRD (17, 31), which FLX also failed to reverse. However, FSL-SIR rats did not exhibit significantly raised plasma inflammatory cytokine levels, although FLX tended to lower plasma TNF-alpha levels.

**Table 2:** Summary of face, construct and predictive validity of the FSL-SIR model with respect to treatment-resistant depression (TRD).

	**Criteria**	**FSL-SIR**	**Congruent for TRD**
Face validity	Increased body weight		♠♦
	Psychomotor agitation		♠♦
	Depression		♠♦
	Social withdrawal		♠♦
	Social anxiety		♠♦
Construct validity	NE		Low ♦
	5-HT		Low ♦
	DA		Low ♦
	DBH		Low ♦
	CORT		Elevated ♦
	IL-6	Unchanged	▾
	TNF-α	Unchanged	▾
Predictive validity	FLX		Poor response ♦

*Key: ♠ – present in the clinical disorder; ♦ – Congruent with the disorder; ■ – high/strong validity; ▾ – conflicting clinical data. Indications of significance/trends, references, and comparators are detailed in the text*.

The authors note some limitations of the study. Observations such as psychomotor agitation were made that, while congruent with literature, may not be a clear cut distinction between MD and TRD and thus require further study into the broader validation aspects of this new model. SIR is known to produce psychotic-like symptoms while some forms of TRD present with co-occurring psychotic symptoms. It is possible that underlying psychotic-like manifestations are present in FSL-SIR rats, with an improved response perhaps realised with a FLX-olanzapine combination. Indeed, reduced frontocortical DA and reduced plasma DBH allude to this possibility. Such behaviours would not be evident within the range of behavioural tests performed in this paper, while response to an atypical antipsychotic with/without an SSRI is recommended to improve predictive validity. These limitations are addressed in a companion paper to this manuscript where the face and predictive validity of this model are expanded (Mncube et al., 2021, submitted).

In conclusion, exposure of a genetic animal model of MD to post-weaning SIR results in a more intractable depressive-like phenotype as well as changes in TRD-related biomarkers, and variable monoamine data, which indicate treatment resistance to a first line antidepressant.

## Data Availability Statement

The raw data supporting the conclusions of this article will be made available by the authors, without undue reservation.

## Ethics Statement

The animal study was reviewed and approved by AnimCare Animal Research Committee (NHREC reg. no. AREC-130913-015) of the North West University (NWU).

## Author Contributions

KM designed the study, treated the animals and collected the samples, performed behavioural and bioanalytical procedures as well as the statistical analysis, interpreted the results, and prepared the first draft as well as the final version of the manuscript. MM advised on the design of the study and on setting up of the social isolation rearing model and social interaction protocols, co-supervised and assisted with the statistical analysis. BH devised the concept of the study, advised on the design of the study, supervised KM, assisted in interpreting the results, co-wrote the manuscript, and prepared it for submission. All authors contributed to the article and approved the submitted version.

## Funding

Research reported in this publication was supported by the South African Medical Research Council (BH). KM gratefully acknowledges the financial assistance of the National Research Foundation of South Africa.

## Author Disclaimer

KM acknowledges that opinions, findings and conclusions or recommendations expressed in any publication generated by NRF supported research are those of the authors, and that the NRF accepts no liability whatsoever in this regard. The earlier-mentioned funders had no other involvement in the study.

## Conflict of Interest

With respect to this work, the authors declare that over the past 3 years, BH has participated in advisory boards and received honoraria from Servier and Lundbeck, and has received research funding from Servier, Lundbeck, and HG & H Pharma. BH would like to declare that this paper is a contribution to a Research Topic entitled “Animal Models in Psychiatry: Translating Animal Behavior to an Improved Understanding and Treatment of Psychiatric Disorders”, where he is a Topic Editor. The authors declare that, except for income from the primary employer and research funding to BH from the below-mentioned organisations and agencies, no financial support or compensation has been received from any individual or corporate entity over the past 3 years for research or professional services, and there are no personal financial holdings that could be perceived as constituting a potential conflict of interest. The remaining authors declare that the research was conducted in the absence of any commercial or financial relationships that could be construed as a potential conflict of interest.

## Publisher's Note

All claims expressed in this article are solely those of the authors and do not necessarily represent those of their affiliated organizations, or those of the publisher, the editors and the reviewers. Any product that may be evaluated in this article, or claim that may be made by its manufacturer, is not guaranteed or endorsed by the publisher.

## References

[1] Cusin C Peyda S. Treatment-resistant depression. In: Shapero BG Mischoulon D Cusin C. editors. Book Treatment-Resistant Depression. Cham: Springer International Publishing (2019). p. 3–19.

[2] Berlim MT Turecki G. Definition, assessment, and staging of treatment—resistant refractory major depression: a review of current concepts and methods. Can J Psychiatry. (2007) 52:46–54. 10.1177/070674370705200108 17444078

[3] Trivedi MH Rush AJ Wisniewski SR Nierenberg AA Warden D Ritz L. Evaluation of outcomes with citalopram for depression using measurement-based care in STAR^*^D: implications for clinical practice. Am J Psychiatry. (2006) 163:28–40. 10.1176/appi.ajp.163.1.28 16390886

[4] Al-Harbi KS. Treatment-resistant depression: therapeutic trends, challenges, future directions. Patient Prefer Adherence. (2012) 6:369–88. 10.2147/PPA.S29716 22654508 PMC3363299

[5] Rush AJ Trivedi MH Wisniewski SR Nierenberg AA Stewart JW Warden D. Acute and longer-term outcomes in depressed outpatients requiring one or several treatment steps: a STAR^*^D report. Am J Psychiatry. (2006) 163:1905–17. 10.1176/ajp.2006.163.11.1905 17074942

[6] Kornstein SG Schneider RK. Clinical features of treatment-resistant depression. J Clin Psychiatry. (2001) (Suppl. 16):18–25. 11480880

[7] Rosenzweig-Lipson S Beyer CE Hughes ZA Khawaja X Rajarao SJ Malberg JE. Differentiating antidepressants of the future: efficacy and safety. Pharmacol Ther. (2007) 113:134–53. 10.1016/j.pharmthera.2006.07.002 17010443

[8] Nasca C Bigio B Lee FS Young SP Kautz MM Albright A. Acetyl-L-carnitine deficiency in patients with major depressive disorder. Proc Natl Acad Sci USA. (2018) 115:8627–32. 10.1073/pnas.1801609115 30061399 PMC6112703

[9] Takahashi M Shirayama Y Muneoka K Suzuki M Sato K Hashimoto K. Personality traits as risk factors for treatment-resistant depression. PLoS ONE. (2013) 8:e63756. 10.1371/journal.pone.0063756 23717477 PMC3661718

[10] Souery D Oswald P Massat I Bailer U Bollen J Demyttenaere K. Clinical factors associated with treatment resistance in major depressive disorder: results from a European multicenter study. J Clin Psychiatry. (2007) 68:1062–70. 10.4088/JCP.v68n0713 17685743

[11] Cepeda MS Reps J Ryan P. Finding factors that predict treatment-resistant depression: results of a cohort study. Dep Anxiety. (2018) 35:668–73. 10.1002/da.22774 29786922 PMC6055726

[12] Rizvi SJ Grima E Tan M Rotzinger S Lin P McIntyre RS. Treatment-resistant depression in primary care across Canada. Can J Psychiatry. (2014) 59:349–57. 10.1177/070674371405900702 25007419 PMC4086317

[13] Fink M. Separating psychotic depression from nonpsychotic depression is essential to effective treatment. J Affec Dis. (2003) 76:1–3. 10.1016/S0165-0327(02)00175-1 12943927

[14] Bobo WV Shelton RC. Olanzapine and fluoxetine combination therapy for treatment-resistant depression: review of efficacy, safety, and study design issues. Neuro Dis Treat. (2009) 5:369–83. 10.2147/NDT.S5819 19590732 PMC2706569

[15] Hurst M Lamb HM. Fluoxetine. CNS Drugs. (2000) 14:51–80. 10.2165/00023210-200014010-00005

[16] Bymaster FP Zhang W Carter PA Shaw J Chernet E Phebus L. Fluoxetine, but not other selective serotonin uptake inhibitors, increases norepinephrine and dopamine extracellular levels in prefrontal cortex. Psychopharmacology. (2002) 160:353–61. 10.1007/s00213-001-0986-x 11919662

[17] Caldarone BJ Zachariou V King SL. Rodent models of treatment-resistant depression. Eur J Pharmacol. (2015) 753:51–65. 10.1016/j.ejphar.2014.10.063 25460020 PMC4423538

[18] Atmore KH Stein DJ Harvey BH Russell VA Howells FM. Differential effects of social isolation rearing on glutamate- and GABA-stimulated noradrenaline release in the rat prefrontal cortex and hippocampus. Euro Neuropsychopharmacol. (2020) 36:111–20. 10.1016/j.euroneuro.2020.05.007 32553548

[19] Brand SJ Moller M Harvey BH. A review of biomarkers in mood and psychotic disorders: a dissection of clinical vs. Preclinical correlates. Curr Neuropharmacol. (2015) 13:324–68. 10.2174/1570159X13666150307004545 26411964 PMC4812797

[20] Maes M Bosmans E De Jongh R Kenis G Vandoolaeghe E Neels H. Increased serum IL-6 and IL-1 receptor antagonist concentrations in major depression and treatment resistant depression. Cytokine. (1997) 9:853–8. 10.1006/cyto.1997.0238 9367546

[21] Hori H Kunugi H. The efficacy of pramipexole, a dopamine receptor agonist, as an adjunctive treatment in treatment-resistant depression: an open-label trial. Sci World J. (2012) 2012:1–8. 10.1100/2012/372474 22919308 PMC3415165

[22] Wijeratne C Sachdev P. Treatment-resistant depression: critique of current approaches. Aust Psychiatry NZJ. (2008) 42:751–62. 10.1080/00048670802277206 18696279

[23] Schatzberg AF Rothschild AJ. Psychotic (delusional) major depression: should it be included as a distinct syndrome in DSM-IV? Am J Psychiatry. (1992) 149:733–45. 10.1176/ajp.149.6.733 1590491

[24] Caraci F Calabrese F Molteni R Bartova L Dold M Leggio GM. International union of basic and clinical pharmacology CIV: the neurobiology of treatment-resistant depression: from antidepressant classifications to novel pharmacological targets. Pharmacol Rev. (2018) 70:475–504. 10.1124/pr.117.014977 29884653

[25] Coplan JD Gopinath S Abdallah CG Berry BR. A neurobiological hypothesis of treatment-resistant depression – mechanisms for selective serotonin reuptake inhibitor non-efficacy. Front Behav Neurosci. (2014) 8:1–16. 10.3389/fnbeh.2014.00189 24904340 PMC4033019

[26] Maletic V Eramo A Gwin K Offord SJ Duffy RA. The role of norepinephrine and its α-adrenergic receptors in the pathophysiology and treatment of major depressive disorder and schizophrenia: a systematic review. Front Psychiatry. (2017) 8:42. 10.3389/fpsyt.2017.00042 28367128 PMC5355451

[27] Uys MM Shahid M Harvey BH. Therapeutic potential of selectively targeting the α2C-adrenoceptor in cognition, depression, and schizophrenia—new developments and future perspective. Front Psychiatry. (2017) 8:1–23. 10.3389/fpsyt.2017.00144 28855875 PMC5558054

[28] Villas Boas GR Boerngen de Lacerda R Paes MM Gubert P Almeida W Rescia VC. Molecular aspects of depression: a review from neurobiology to treatment. Eur J Pharmacol. (2019) 851:99–121. 10.1016/j.ejphar.2019.02.024 30776369

[29] Overstreet DH Wegener G. The flinders sensitive line rat model of depression−25 years and still producing. Pharmacol Rev. (2013) 65:143–55. 10.1124/pr.111.005397 23319547

[30] Overstreet DH. The flinders sensitive line rats: a genetic animal model of depression. Neurosci Biobehav Rev. (1993) 17:51–68. 10.1016/S0149-7634(05)80230-1 8455816

[31] Willner P Belzung C. Treatment-resistant depression: are animal models of depression fit for purpose? Psychopharmacology. (2015) 232:3473–95. 10.1007/s00213-015-4034-7 26289353

[32] Fone KC Porkess MV. Behavioural and neurochemical effects of post-weaning social isolation in rodents-relevance to developmental neuropsychiatric disorders. Neurosci Biobehav Rev. (2008) 32:1087–102. 10.1016/j.neubiorev.2008.03.003 18423591

[33] Regenass W Moller M Harvey BH. Studies into the anxiolytic actions of agomelatine in social isolation reared rats: role of corticosterone and sex. J Psychopharmacol. (2018) 32:134–45. 10.1177/0269881117735769 29082818

[34] Moller M Du Preez JL Emsley R Harvey BH. Isolation rearing-induced deficits in sensorimotor gating and social interaction in rats are related to cortico-striatal oxidative stress, and reversed by sub-chronic clozapine administration. Eur Neuropsychopharmacol. (2011) 21:471–83. 10.1016/j.euroneuro.2010.09.006 20965701

[35] Pereira VS Joca SRL Harvey BH Elfving B Wegener G. Esketamine and rapastinel, but not imipramine, have antidepressant-like effect in a treatment-resistant animal model of depression. Acta Neuropsychiatr. (2019) 31:258–65. 10.1017/neu.2019.25 31230597

[36] Brand SJ Harvey BH. Exploring a post-traumatic stress disorder paradigm in flinders sensitive line rats to model treatment-resistant depression I: bio-behavioural validation and response to imipramine. Acta Neuropsychiatr. (2017) 29:193–206. 10.1017/neu.2016.44 27573792

[37] Kin K Yasuhara T Kawauchi S Kameda M Hosomoto K Tomita Y. Lithium counteracts depressive behavior and augments the treatment effect of selective serotonin reuptake inhibitor in treatment-resistant depressed rats. Brain Res. (2019) 1717:52–9. 10.1016/j.brainres.2019.04.001 30953607

[38] Marchetti L Lauria M Caberlotto L Musazzi L Popoli M Mathé AA. Gene expression signature of antidepressant treatment response/non-response in flinders sensitive line rats subjected to maternal separation. Eur Neuropsychopharmacol. (2020) 31:69–85. 10.1016/j.euroneuro.2019.11.004 31813757

[39] Swanepoel T Moller M Harvey BH. N-acetyl cysteine reverses bio-behavioural changes induced by prenatal inflammation, adolescent methamphetamine exposure and combined challenges. Psychopharmacology. (2018) 235:351–68. 10.1007/s00213-017-4776-5 29116368

[40] Vargas J Junco M Gomez C Lajud N. Early life stress increases metabolic risk, hpa axis reactivity, and depressive-like behavior when combined with postweaning social isolation in rats. PLoS ONE. (2016) 11:e0162665–. 10.1371/journal.pone.0162665 27611197 PMC5017766

[41] Walker DM Cunningham AM Gregory JK Nestler EJ. Long-term behavioral effects of post-weaning social isolation in males and females. Front Behav Neurosci. (2019) 13:1–20. 10.3389/fnbeh.2019.00066 31031604 PMC6470390

[42] Weiss IC Pryce CR Jongen-Rêlo AL Nanz-Bahr NI Feldon J. Effect of social isolation on stress-related behavioural and neuroendocrine state in the rat. Behav Brain Res. (2004) 152:279–95. 10.1016/j.bbr.2003.10.015 15196796

[43] Moller M Du Preez JL Viljoen FP Berk M Emsley R Harvey BH. Social isolation rearing induces mitochondrial, immunological, neurochemical and behavioural deficits in rats, and is reversed by clozapine or N-acetyl cysteine. Brain Behav Immun. (2013) 30:156–67. 10.1016/j.bbi.2012.12.011 23270677

[44] Uys M Shahid M Sallinen J Dreyer W Cockeran M Harvey BH. The alpha2C-adrenoceptor antagonist, ORM-10921, has antipsychotic-like effects in social isolation reared rats and bolsters the response to haloperidol. Prog Neuropsychopharmacol Biol Psychiatry. (2016) 71:108–16. 10.1016/j.pnpbp.2016.07.002 27381554

[45] Mokoena ML Harvey BH Viljoen F Ellis SM Brink CB. Ozone exposure of flinders sensitive line rats is a rodent translational model of neurobiological oxidative stress with relevance for depression and antidepressant response. Psychopharmacology. (2015) 232:2921–38. 10.1007/s00213-015-3928-8 25877744

[46] Detke MJ Rickels M Lucki I. Active behaviors in the rat forced swimming test differentially produced by serotonergic and noradrenergic antidepressants. Psychopharmacolog. (1995) 121:66–72. 10.1007/BF02245592 8539342

[47] Zhang W Perry KW Wong DT Potts BD Bao J Tollefson GD. Synergistic effects of olanzapine and other antipsychotic agents in combination with fluoxetine on norepinephrine and dopamine release in rat prefrontal cortex. Neuropsychopharmacology. (2000) 23:250–62. 10.1016/S0893-133X(00)00119-6 10942849

[48] Łojko D Rybakowski JK. Atypical depression: current perspectives. Neuro Dis Treat. (2017) 13:2447. 10.2147/NDT.S147317 29033570 PMC5614762

[49] Sherif F Oreland L. Effect of the GABA-transaminase inhibitor vigabatrin on exploratory behaviour in socially isolated rats. Behav Brain Res. (1995) 72:135–40. 10.1016/0166-4328(96)00047-2 8788866

[50] Schoeman JC Steyn SF Harvey BH Brink CB. Long-lasting effects of fluoxetine and/or exercise augmentation on bio-behavioural markers of depression in pre-pubertal stress sensitive rats. Behav Brain Res. (2017) 323:86–99. 10.1016/j.bbr.2017.01.043 28143768

[51] Oberholzer I Moller M Holland B Dean OM Berk M Harvey BH. Garcinia mangostana linn displays antidepressant-like and pro-cognitive effects in a genetic animal model of depression: a bio-behavioral study in the flinders sensitive line rat. Metab Brain Dis. (2018) 33:467–80. 10.1007/s11011-017-0144-8 29101602

[52] File SE Seth P. A review of 25 years of the social interaction test. Eur J Pharmacol. (2003) 463:35–53. 10.1016/S0014-2999(03)01273-1 12600701

[53] Kaidanovich-Beilin O Lipina T Vukobradovic I Roder J Woodgett JR. Assessment of social interaction behaviors. J Vis Exp. (2011) e2473:1–6. 10.3791/2473 21403628 PMC3197404

[54] Ramos A Berton O Mormède P Chaouloff F. A multiple-test study of anxiety-related behaviours in six inbred rat strains. Behav Brain Res. (1997) 85:57–69. 10.1016/S0166-4328(96)00164-7 9095342

[55] Barnett SA. An analysis of social behaviour in wild rats. Proc Zool Soc Lond. (1958) 130:107–52. 10.1111/j.1096-3642.1958.tb00565.x

[56] Brain PF McAllister KH Walmsley S. Drug Effects on Social Behavior. Book Drug Effects on Social Behavior. Totowa, NJ: Humana Press (1989) p. 687–739.

[57] Wilson CA Koenig JI. Social interaction and social withdrawal in rodents as readouts for investigating the negative symptoms of schizophrenia. Eur Neuropsychopharmacol. (2014) 24:759–73. 10.1016/j.euroneuro.2013.11.008 24342774 PMC4481734

[58] del Angel Ortiz R Contreras CM Gutiérrez-Garcia AG González MFM. Social interaction test between a rat and a robot: a pilot study. Int J Adv Robot Syst. (2016) 13:4. 10.5772/62015

[59] Möller M Du Preez JL Viljoen FP Berk M Harvey BH. N-acetyl cysteine reverses social isolation rearing induced changes in cortico-striatal monoamines in rats. Metabolic Brain Disease. (2013) 28:687–96. 10.1007/s11011-013-9433-z 24000072

[60] Viljoen FP du Preez JL Wessels JC Aucamp ME. HPLC electrochemical detection and quantification of monoamines and their metabolites in rat brain tissue samples. Pharmazie. (2018) 73:563–9. 10.1691/ph.2018.8099 30223919

[61] Brand SJ Harvey BH. Exploring a post-traumatic stress disorder paradigm in flinders sensitive line rats to model treatment-resistant depression II: response to antidepressant augmentation strategies. Acta Neuropsychiatr. (2017) 29:207–21. 10.1017/neu.2016.50 27692010

[62] Sawilowsky SS. New effect size rules of thumb. J Mod Appl Stat Meth. (2009) 8:26. 10.22237/jmasm/1257035100

[63] Willner P. The chronic mild stress (CMS) model of depression: history, evaluation and usage. Neurobiol Stress. (2017) 6:78–93. 10.1016/j.ynstr.2016.08.002 28229111 PMC5314424

[64] Kirkedal C Elfving B Müller HK Moreira FA Bindila L Lutz B. Hemisphere-dependent endocannabinoid system activity in prefrontal cortex and hippocampus of the flinders sensitive line rodent model of depression. Neurochem Int. (2019) 125:7–15. 10.1016/j.neuint.2019.01.023 30716357

[65] Liao L Wu Z Mellor D Peng D Zhang C Xu J. Subtypes of treatment-resistant depression determined by a latent class analysis in a chinese clinical population. J Affect Dis. (2019) 249:82–9. 10.1016/j.jad.2019.02.005 30763799

[66] Silva MTA Alves C Santarem E. Anxiogenic-like effect of acute and chronic fluoxetine on rats tested on the elevated plus-maze. Brazil J Med Biol Res. (1999) 32:333–9. 10.1590/S0100-879X1999000300014 10347793

[67] Overstreet DH Friedman E Mathé AA Yadid G. The flinders sensitive line rat: a selectively bred putative animal model of depression. Neurosci Biobehav Rev. (2005) 29:739–59. 10.1016/j.neubiorev.2005.03.015 15925699

[68] Malhi GS Das P Mannie Z Irwin L. Treatment-resistant depression: problematic illness or a problem in our approach? Brit J Psychiatry. (2019) 214:1–3. 10.1192/bjp.2018.246 30565539

[69] Arndt DL Peterson CJ Cain ME. Differential rearing alters forced swim test behavior, fluoxetine efficacy, and post-test weight gain in male rats. PLoS ONE. (2015) 10:e0131709. 10.1371/journal.pone.0131709 26154768 PMC4496081

[70] Anyan J Amir S. Too depressed to swim or too afraid to stop? A reinterpretation of the forced swim test as a measure of anxiety-like behavior. Neuropsychopharmacology. (2018) 43:931–3. 10.1038/npp.2017.260 29210364 PMC5854810

[71] Andrews PW Bharwani A Lee KR Fox M Thomson JA. Is serotonin an upper or a downer? The evolution of the serotonergic system and its role in depression and the antidepressant response. Neurosci Biobehav Rev. (2015) 51:164–88. 10.1016/j.neubiorev.2015.01.018 25625874

[72] Harvey BH Naciti C Brand L Stein DJ. Serotonin and stress: protective or malevolent actions in the biobehavioral response to repeated trauma? Ann N Y Acad Sci. (2004) 1032:267–72. 10.1196/annals.1314.035 15677425

[73] Gournellis R Tournikioti K Touloumi G Thomadakis C Michalopoulou PG Christodoulou C. Psychotic (delusional) depression and suicidal attempts: a systematic review and meta-analysis. Acta Psychiatrica Scandinavica. (2018) 137:18–29. 10.1111/acps.12826 29178463

[74] Perugi G Pacchiarotti I Mainardi C Verdolini N Menculini G Barbuti M. Patterns of response to antidepressants in major depressive disorder: drug resistance or worsening of depression are associated with a bipolar diathesis. Eur Neuropsychopharmacol. (2019) 29:825–34. 10.1016/j.euroneuro.2019.06.001 31227264

[75] Liebenberg N Harvey BH Brand L Wegener G Brink CB. Chronic treatment with the phosphodiesterase type 5 inhibitors sildenafil and tadalafil display anxiolytic effects in flinders sensitive line rats. Metab Brain Dis. (2012) 27:337–40. 10.1007/s11011-012-9284-z 22359075

[76] American Psychiatric Association. Diagnostic and Statistical Manual of Mental Disorders: DSM-5. 5th ed. Washington, DC: American Psychiatric Association (2013).

[77] Elliott BM Grunberg NE. Effects of social and physical enrichment on open field activity differ in male and female sprague-dawley rats. Behav Brain Res. (2005) 165:187–96. 10.1016/j.bbr.2005.06.025 16112757

[78] Samuels BA Leonardo ED Gadient R Williams A Zhou J David DJ. Modeling treatment-resistant depression. Neuropharmacol. (2011) 61:408–13. 10.1016/j.neuropharm.2011.02.017 21356220 PMC3110541

[79] Dulawa SC Holick KA Gundersen B Hen R. Effects of chronic fluoxetine in animal models of anxiety and depression. Neuropsychopharmacol. (2004) 29:1321. 10.1038/sj.npp.1300433 15085085

[80] Farhan M Haleem DJ. Anxiolytic profile of fluoxetine as monitored following repeated administration in animal rat model of chronic mild stress. Saudi Pharm J. (2016) 24:571–8. 10.1016/j.jsps.2015.03.006 27752230 PMC5059824

[81] Smolders I Clinckers R Meurs A De Bundel D Portelli J Ebinger G. Direct enhancement of hippocampal dopamine or serotonin levels as a pharmacodynamic measure of combined antidepressant–anticonvulsant action. Neuropharmacol. (2008) 54:1017–28. 10.1016/j.neuropharm.2008.02.006 18378264

[82] Ahrold TK Meston CM. Effects of SNS activation on SSRI-induced sexual side effects differ by SSRI. J Sex Marital Ther. (2009) 35:311–9. 10.1080/00926230902851322 19466669 PMC4426856

[83] Yohn CN Gergues MM Samuels BA. The role of 5-HT receptors in depression. Molecular brain. (2017) 10:28–8. 10.1186/s13041-017-0306-y 28646910 PMC5483313

[84] Amidfar M Colic L Walter M Kim Y-K. Biomarkers of major depression related to serotonin receptors. Curr Psychiatry Rev. (2018) 14:239–44. 10.2174/157340051466618101611574723547754

[85] Cottingham C Wang Q. α2 adrenergic receptor dysregulation in depressive disorders: Implications for the neurobiology of depression and antidepressant therapy. Neurosci Biobehav Rev. (2012) 36:2214–25. 10.1016/j.neubiorev.2012.07.011 22910678 PMC3508310

[86] Kovacevic T Skelin I Diksic M. Chronic fluoxetine treatment has a larger effect on the density of a serotonin transporter in the flinders sensitive line (FSL) rat model of depression than in normal rats. Synapse. (2010) 64:231–40. 10.1002/syn.20721 19924693

[87] Zangen A Overstreet DH Yadid G. High serotonin and 5-hydroxyindoleacetic acid levels in limbic brain regions in a rat model of depression: normalization by chronic antidepressant treatment. J Neurochem. (1997) 69:2477–83. 10.1046/j.1471-4159.1997.69062477.x 9375680

[88] Kuramochi M Nakamura S. Effects of postnatal isolation rearing and antidepressant treatment on the density of serotonergic and noradrenergic axons and depressive behavior in rats. Neuroscience. (2009) 163:448–55. 10.1016/j.neuroscience.2009.06.017 19524023

[89] Muchimapura S Mason R Marsden CA. Effect of isolation rearing on pre- and post-synaptic serotonergic function in the rat dorsal hippocampus. Synapse. (2003) 47:209–17. 10.1002/syn.10167 12494403

[90] Matsuzaki H Izumi T Horinouchi T Boku S Inoue T Yamaguchi T. Juvenile stress attenuates the dorsal hippocampal postsynaptic 5-HT1A receptor function in adult rats. Psychopharmacology. (2011) 214:329–37. 10.1007/s00213-010-1987-4 20714708

[91] Willner P Scheel-Krüger J Belzung C. The neurobiology of depression and antidepressant action. Neurosci Biobehav Rev. (2013) 37(10, Part 1):2331–71. 10.1016/j.neubiorev.2012.12.007 23261405

[92] Cryan JF Slattery DA. Animal models of mood disorders: recent developments. Curr Opin Psychiatry. (2007) 20:1–7. 10.1097/YCO.0b013e3280117733 17143074

[93] Petersen T Papakostas GI Mahal Y Guyker WM Beaumont EC Alpert JE. Psychosocial functioning in patients with treatment resistant depression. Eur Psychiatry. (2004) 19:196–201. 10.1016/j.eurpsy.2003.11.006 15196600

[94] Tillmann S Awwad HM Eskelund AR Treccani G Geisel J Wegener G. Probiotics affect one-carbon metabolites and catecholamines in a genetic rat model of depression. Mol Nutr Food Res. (2018) 62:e1701070–e1701070. 10.1002/mnfr.201701070 29453804 PMC5900923

[95] Kobayashi K Haneda E Higuchi M Suhara T Suzuki H. Chronic fluoxetine selectively upregulates dopamine D1-like receptors in the hippocampus. Neuropsychopharmacol. (2012) 37:1500–8. 10.1038/npp.2011.335 22278095 PMC3327854

[96] Hess C Reif A Strobel A Boreatti-Hümmer A Heine M Lesch K-P. A functional dopamine-β-hydroxylase gene promoter polymorphism is associated with impulsive personality styles, but not with affective disorders. J Neural Transm. (2009) 116:121–30. 10.1007/s00702-008-0138-0 18982239

[97] Meltzer HY Cho HW Carroll BJ. Serum dopamine-βhydroxylase activity in the affective psychoses and schizophrenia: decreased activity in unipolar psychotically depressed patients. Arch Gen Psychiatry. (1976) 33:585–91. 10.1001/archpsyc.1976.01770050047007 1267575

[98] Meyers BS Alexopoulos GS Kakuma T Tirumalasetti F Gabriele M Alpert S. Decreased dopamine beta-hydroxylase activity in unipolar geriatric delusional depression. Biol Psychiatry. (1999) 45:448–52. 10.1016/S0006-3223(98)00085-7 10071716

[99] Serova L Sabban EL Zangen A Overstreet DH Yadid G. Altered gene expression for catecholamine biosynthetic enzymes and stress response in rat genetic model of depression. Mol Brain Res. (1998) 63:133–8. 10.1016/S0169-328X(98)00270-8 9838081

[100] Pariante CM Thomas SA Lovestone S Makoff A Kerwin RW. Do antidepressants regulate how cortisol affects the brain? Psychoneuroendocrinology. (2004) 29:423–47. 10.1016/j.psyneuen.2003.10.009 14749091

[101] Heydendael W Jacobson L. Widespread hypothalamic–pituitary–adrenocortical axis-relevant and mood-relevant effects of chronic fluoxetine treatment on glucocorticoid receptor gene expression in mice. Eur J Neurosci. (2010) 31:892–902. 10.1111/j.1460-9568.2010.07131.x 20374287

[102] Carboni L Becchi S Piubelli C Mallei A Giambelli R Razzoli M. Early-life stress and antidepressants modulate peripheral biomarkers in a gene-environment rat model of depression. Prog Neuropsychopharmacol Biol Psychiatry. (2010) 34:1037–48. 10.1016/j.pnpbp.2010.05.019 20580919

[103] Perlman K Benrimoh D Israel S Rollins C Brown E Tunteng JF. A systematic meta-review of predictors of antidepressant treatment outcome in major depressive disorder. J Affect Disord. (2019) 243:503–15. 10.1016/j.jad.2018.09.067 30286415

[104] Roumestan C Michel A Bichon F Portet K Detoc M Henriquet C. Anti-inflammatory properties of desipramine and fluoxetine. Res Res. (2007) 8:35. 10.1186/1465-9921-8-35 17477857 PMC1876225

[105] Bianchi M Sacerdote P Panerai P. Fluoxetine reduces inflammatory edema in the rat: involvement of the pituitary-adrenal axis. Eur J Pharmacol. (1994) 263:81–84. 10.1016/0014-2999(94)90526-6 7821365

[106] Goh J.-Y., O'Sullivan SE, Shortall SE, Zordan N, Piccinini AM, et al. Gestational poly(I:C) attenuates, not exacerbates, the behavioral, cytokine and mTOR changes caused by isolation rearing in a rat ‘dual-hit’ model for neurodevelopmental disorders. Brain Behav Immun. (2020) 89:100–17. 10.1016/j.bbi.2020.05.076 32485291

[107] Daskalakis NP Oitzl MS Schächinger H Champagne DL Ronald de Kloet E. Testing the cumulative stress and mismatch hypotheses of psychopathology in a rat model of early-life adversity. Physiol Behav. (2012) 106:707–21. 10.1016/j.physbeh.2012.01.015 22306534

[108] Seery MD Leo RJ Lupien SP Kondrak CL Almonte JL. An upside to adversity?: moderate cumulative lifetime adversity is associated with resilient responses in the face of controlled stressors. Psychol Sci. (2013) 24:1181–9. 10.1177/0956797612469210 23673992

[109] Keller J Schatzberg AF Maj M. Current issues in the classification of psychotic major depression. Schizophr Bull. (2007) 33:877–85. 10.1093/schbul/sbm065 17548842 PMC2632329

[110] Schrijvers D Hulstijn W Sabbe BGC. Psychomotor symptoms in depression: a diagnostic, pathophysiological and therapeutic tool. J Affec Dis. (2008) 109:1–20. 10.1016/j.jad.2007.10.019 18082896

[111] Schatzberg AF Keller J Tennakoon L Lembke A Williams G Kraemer FB. HPA axis genetic variation, cortisol and psychosis in major depression. Mol Psychiatry. (2014) 19:220–7. 10.1038/mp.2013.129 24166410 PMC4339288

